# Functional Tooth Units, Oral Disease Status and Dietary Quality in a Representative US Population

**DOI:** 10.1111/jcpe.70165

**Published:** 2026-07-13

**Authors:** Yu Xie, Mengning Bi, Xiaoyu Yu, Jie Shen, Hairui Li, Yuan Li, Andrea Roccuzzo, Changzheng Yuan, Maurizio S. Tonetti

**Affiliations:** ^1^ Shanghai PerioImplant Innovation Center and Oral Biomedical Intelligence Technology (ORAL‐BIT) Laboratory, Institute for Oral, Craniofacial and Sensory Research, Ninth People's Hospital Shanghai Jiao Tong University School of Medicine Shanghai China; ^2^ National Clinical Research Center of Oral Diseases and National Center of Stomatology, College of Stomatology Shanghai Jiao Tong University School of Medicine Shanghai China; ^3^ School of Public Health, The Second Affiliated Hospital Zhejiang University School of Medicine Hangzhou China; ^4^ Department of Nutrition Harvard T.H. Chan School of Public Health Boston Massachusetts USA; ^5^ European Research Group on Periodontology (ERGOPerio) Genova Italy

**Keywords:** caries, diet, functional tooth units, NHANES, nutrition, periodontitis, tooth loss

## Abstract

**Aim:**

To investigate how functional tooth units (FTUs) and oral conditions are associated with dietary quality through multidimensional measures within a unified analytic framework.

**Materials and Methods:**

Data were drawn from 6291 adults in the 2011–2014 National Health and Nutrition Examination Survey. Dentition status was assessed using FTUs and tooth count, and oral disease status was classified based on both untreated caries and periodontitis. Dietary outcomes derived from two 24‐h dietary recalls included the Healthy Eating Index‐2020 (HEI‐2020), Dietary Approaches to Stop Hypertension index (DASH), alternate Mediterranean diet score (MED), Dietary Inflammation Index (DII), food groups and nutrient intakes. Distributional differences and associations between oral conditions and dietary outcomes were assessed using survey‐weighted analyses.

**Results:**

Participants with adequate FTUs had higher dietary quality than participants with impaired FTUs or edentulous participants. In fully adjusted models, each additional FTU was associated with higher HEI‐2020 scores (β = 0.41, 95% CI: 0.29–0.54), lower DII scores (β = −0.06, 95% CI: −0.09 to −0.04) and more reported food items (β = 0.09, 95% CI: 0.04–0.14). Among dentate adults, untreated caries status was linked to less favourable dietary profiles after FTUs adjustment. In disease extent analyses, Decay% was associated with higher dietary quality indices than Decay + Filling% or periodontal parameters. After full adjustment, only Decay% remained significantly associated with added sugar intake in the analyses for foods and nutrients. In addition, oral conditions were significantly associated with overall food or nutrient variation, whereas sociodemographic factors accounted for a larger proportion of overall variation.

**Conclusions:**

Oral conditions show distinct associations with dietary quality. Impaired FTUs are linked to poorer dietary quality; coexisting caries and periodontitis are associated with the least favourable dietary quality. Oral conditions may help identify adults with less favourable dietary profiles, although sociodemographic factors explained greater overall dietary variation.

## 1 Introduction

High‐quality dietary intake is a fundamental determinant of health, supporting immunity (Munteanu and Schwartz 2022), metabolic function (Arneth 2025) and tissue homeostasis (Shenkin 2006) and preventing disease and functional decline. An expanding body of evidence has linked dietary patterns to primary systemic and oral health outcomes (Delgado‐Lista et al. 2022; Duggan et al. 2023; Mazurkiewicz et al. 2023; Shi et al. 2025), underscoring diet as an important and modifiable risk factor with broad public health implications.

Oral conditions have been associated with overall dietary quality and nutritional adequacy. Compromised oral health may co‐occur with differences in food selection and nutritional quality (Moynihan and Petersen 2004; Nowjack‐Raymer and Sheiham 2003; Sheiham et al. 2001). Although several studies associate greater tooth loss with lower intake of fruits, vegetables and fibre and with higher cholesterol consumption, a systematic review concluded that the overall longitudinal evidence is mixed and of low quality (Gaewkhiew et al. 2017). Compared with simple tooth counts, functional tooth units (FTUs), which account for both the number and the functional arrangement of teeth, provide a more accurate measure of masticatory capacity (Hildebrandt et al. 1997). However, studies on FTUs and dietary intake remain few and have largely focused on older adults or on selected dietary outcomes, highlighting the need for broader population‐based research (Hildebrandt et al. 1997; Milledge et al. 2021).

Dental caries and periodontitis may also be associated with dietary profiles, but potentially through different dietary correlates. These relationships are likely bidirectional: dietary intake may be associated with oral disease development or progression, while oral diseases may co‐occur with altered food choice and nutrient intake. However, evidence on diet and oral health remains fragmented: masticatory function, periodontitis and dental caries have typically been examined separately, often in populations with mixed levels of masticatory function, making it difficult to isolate disease‐specific associations and to represent individuals with coexisting conditions (Au Yeung et al. 2024; A. Li et al. 2021; X. Y. Li et al. 2022). Periodontitis and dental caries are also rarely evaluated side by side within a unified analytic framework, and dietary assessment has often relied on narrow indicators, focusing on a single dietary pattern or isolated nutrients rather than the multidimensional nature of diet.

Against this background, the present study sought to provide a higher level overview of the relationship between oral health and dietary quality by jointly considering three key dimensions—FTUs, periodontitis and dental caries—within a single analytic framework. Using data from a nationally representative sample of US adults in the 2011–2012 and 2013–2014 National Health and Nutrition Examination Survey (NHANES) cycles, we aimed to (1) examine associations between FTUs and dietary quality; (2) evaluate associations of the presence and clinical extent of dental caries and periodontitis with dietary quality, before and after accounting for FTUs; and (3) contextualise the variance in overall dietary profiles explained by oral conditions relative to demographic, lifestyle, health‐related and socioeconomic factors. We hypothesised that fewer FTUs and oral disease subgroups would be associated with less favourable dietary profiles.

## 2 Methods

### 2.1 Study Design and Population

This study used data from the 2011–2012 and 2013–2014 NHANES cycles. NHANES is a nationally representative, cross‐sectional survey of the US non‐institutionalised civilian population, conducted in 2‐year cycles using a complex multistage probability sampling design. Data are collected via standardised in‐home interviews and clinical examinations performed at mobile examination centres (MECs). Detailed protocols are available at the NHANES website (https://www.cdc.gov/nchs/nhanes/index.htm).

Based on the study objectives, participants were excluded if they were (i) younger than 18 years, (ii) pregnant, (iii) lacking complete full‐mouth oral examination data or (iv) had incomplete or unreliable two 24‐h dietary recalls. The flow diagram of participant inclusion and exclusion is shown in Figure S1.

### 2.2 Assessment of Dentition Status

Dentition status was assessed primarily using FTUs (Ueno et al. 2008) and sensitivity analysis using tooth count.

FTUs were defined as pairs of opposing posterior teeth including natural teeth as well as implant‐supported and fixed prostheses. As direct occlusal contact data were unavailable in NHANES, FTUs were interpreted as potential posterior occlusal support (Wu et al. 2024). One pair of opposing premolars was counted as one FTU and one pair of opposing molars as two FTUs, whereas third molars were excluded from the assessment (theoretical range: 0–12 FTUs). Previous studies had suggested that ≥ 8 FTUs are generally sufficient for adequate masticatory function (van der Bilt 2011). Accordingly, participants were classified as having an adequate number of FTUs (FTUs ≥ 8), impaired FTUs (FTUs = 0–7, with tooth count > 0) or edentulous (tooth count = 0).

Tooth count included the numbers of natural teeth as well as implant‐supported and fixed prostheses. For sensitivity analyses, participants were grouped as having adequate numbers of teeth (tooth count ≥ 20), numbers of impaired teeth (tooth count = 1–19) or edentulous (tooth count = 0).

### 2.3 Identification of Oral Disease Status

Oral examinations were conducted at MECs. Periodontal status was determined using the Centers for Disease Control and Prevention/American Academy of Periodontology (CDC‐AAP) case definitions (Eke et al. 2012), based on full‐mouth measurements of probing depth (PD), gingival recession and clinical attachment loss (CAL). Coronal decay and restorations were assessed at the tooth‐surface level using a dental explorer. Untreated caries was defined by untreated decay on ≥ 1 tooth surface.

Dentate participants were classified into four mutually exclusive oral disease subgroups based on periodontitis and untreated caries status: no untreated caries/periodontitis; untreated caries‐only; periodontitis‐only; and coexisting untreated caries and periodontitis.

### 2.4 Assessment of Dietary Intake Variables

Dietary data were obtained from two non‐consecutive 24‐h recalls, and the average of Day‐1 and Day‐2 intakes was used to calculate dietary indices, food patterns and nutrient intakes. Dietary indices were computed using the ‘dietaryindex’ R package (Zhan et al. 2024), including healthy eating index‐2020 (HEI‐2020) (Kemp et al. 2022; Kennedy et al. 1995), dietary approaches to stop hypertension index (DASH) (Appel et al. 1997), alternate Mediterranean diet (MED) score (de Koning and Anand 2004) and dietary inflammation index (DII) (Shivappa et al. 2014). Concepts, components and scoring interpretation of dietary indices are provided in Table 1.

**TABLE 1:** Conceptual targets, components and scoring methods of dietary indices.

Dietary Index	Conceptual target	Components contributing to higher index scores	Components contributing to lower index scores	Score interpretation	Data source/calculation
HEI2020	Adherence to 2020–2025 Dietary Guidelines for Americans Assess overall diet quality	Higher intake of: total fruits; whole fruits; total vegetables; greens and beans; total protein foods; seafood and plant proteins; whole grains; dairy; unsaturated to saturated fatty acids ratio	Higher intake of: refined grains; sodium; added sugars; saturated fat	Higher = greater alignment with recommended dietary pattern	Calculated using dietaryindex R package
DASH	Adherence to the Dietary Approaches to Stop Hypertension dietary pattern Assess cardiometabolic‐oriented healthy diet	Higher intake of: fruits and fruit juice; vegetables; nuts, legumes, and soy; whole grains; low‐fat dairy	Higher intake of: sodium; red and processed meats; sugar‐sweetened beverages or beverage‐derived added sugars	Higher = better DASH‐style dietary adherence	Calculated using dietaryindex R package
MED	Adherence to Mediterranean‐style diet Assess plant‐based, unsaturated‐fat‐rich dietary pattern	Higher intake of: fruits and fruit juice; vegetables; whole grains; legumes and soy; nuts; fish; monounsaturated‐to‐saturated fat ratio; moderate alcohol intake	Higher intake of: red and processed meats	Higher = better Mediterranean diet adherence	Calculated using dietaryindex R package
DII	Literature‐derived inflammatory potential of diet Assess pro‐ vs. anti‐inflammatory dietary exposure	Components with pro‐inflammatory weights, include: vitamin B12, carbohydrate, cholesterol, total energy intake, total fat, iron, protein, saturated fat	Components with anti‐inflammatory weights, include: alcohol, vitamin B6, beta‐carotene, caffeine, fibre, folic acid, magnesium, monounsaturated fatty acids, niacin, n‐3 fatty acids, n‐6 fatty acids, polyunsaturated fatty acids, riboflavin, selenium, thiamin, vitamin A, vitamin C, vitamin D, vitamin E, zinc	Higher = more pro‐inflammatory and less favourable dietary profile Lower = more anti‐inflammatory and more favourable dietary profile	Calculated using dietaryindex R package

*Note:* HEI‐2020, DASH, MED, and DII are previously established dietary indices. Higher HEI‐2020, DASH and MED scores indicate more favourable dietary quality, whereas higher DII scores indicate a more pro‐inflammatory dietary profile. All participants included in this study had two reliable 24‐h dietary recalls. Each dietary index was calculated separately for each recall day and then averaged across the 2 days.

Abbreviations: DASH, dietary approaches to stop hypertension index; DII, dietary inflammatory index; HEI‐2020, healthy eating index 2020; MED, alternate Mediterranean diet score.

Thirty‐seven standardised food pattern variables were derived from reported individual foods using the Food Patterns Equivalents Database (FPED). The full list of food patterns and nutrients used in this study is provided in Table S2.

### 2.5 Statistical Analysis

All analyses accounted for NHANES's complex survey design. Two‐day dietary recall weights (WTDR2D) were divided by 2 for pooling two cycles and used as sampling weights, following NHANES Analytic Guidelines (Chen et al. 2018).

Food and nutrient intake variables were energy‐adjusted per 1000 kcal before analysis.

Continuous variables are presented as survey‐weighted medians and interquartile ranges (IQR) to account for the non‐normal distribution of several variables. Categorical variables are presented as unweighted counts and survey‐weighted percentages. Statistical differences were tested using survey‐weighted Kruskal–Wallis tests for continuous variables and survey‐weighted chi‐square tests for categorical variables.

Principal component analysis (PCA) was performed on *z*‐score‐standardised food patterns and nutrient intakes, respectively, to explore overall group differences and identify major contributors. Variance in overall food or nutrient intake, explained by oral conditions, demographic factors, lifestyle factors, health status and socioeconomic factors, was estimated using permutational multivariate analysis of variance (PERMANOVA).

Survey‐weighted linear regression was used to assess associations between dentition status, oral disease extents (Decay%, Decay + Filling%, PD ≥ 4 mm %, CAL ≥ 1 mm %, and CAL ≥ 3 mm %) and dietary intake variables. Different models were fitted with covariate adjustment for FTUs, demographics, lifestyle, health status and socioeconomic factors; model details are provided in the corresponding figure legends. Dietary indices were analysed using raw scores to preserve interpretability, whereas food and nutrient intakes were standardised to *z*‐score before modelling to improve comparability across variables with different units and scales. Regression coefficients for dietary indices represent associations in the original index score, whereas coefficients for foods and nutrients represent differences in standard deviation units. Results are interpreted as adjusted survey‐weighted associations rather than evidence of normality or causality.

Participants with missing covariate data were excluded from the corresponding linear regression model. The Benjamini–Hochberg adjustment was applied to control the false discovery rate in multiple testing. All tests were two‐sided with α = 0.05. Statistical analyses were performed in R 4.4.1 (http://www.R‐project.org, The R Foundation).

## 3 Results

### 3.1 Participant Selection and Characteristics of Included and Excluded Participants

A total of 19,931 individuals from the NHANES 2011–2012 and 2013–2014 cycles were initially screened. After excluding participants who were < 18 years of age, pregnant or with incomplete/unreliable oral examination or dietary recall data, 6291 adults were included in the final analytical sample (Figure S1).

Compared with the excluded adults, the included participants were older, had a higher income–poverty ratio and BMI and differed in race/ethnicity and education level, whereas sex distribution was comparable (Table S1).

Participant characteristics by FTU category are shown in Table 2. Among the 6291 participants, 553 were edentulous, 2174 had impaired FTUs (0–7) and 3564 had adequate FTUs (8–12). Participants in lower FTU categories were older, had lower socioeconomic position, lower energy intake and higher BMI, and more frequently reported smoking, hypertension and diabetes, whereas the sex distribution was comparable across groups.

**TABLE 2:** Characteristics of 6219 participants included in the present study classified by the numbers of functional tooth units.

Variables	All (*n* = 6291)	Edentulous	Impaired nif‐FTUs	Adequate nif‐FTUs	*p* value
		(*n* = 553)	(*n* = 2174)	(*n* = 3564)	
Age (year)	51.0 (40.0–62.0)	68.0 (57.0–76.0)	57.0 (47.0–65.0)	47.0 (38.0–58.0)	**< 0.001**
Sex					0.676
Male	3044 (48.6%)	282 (50.4%)	1046 (49.1%)	1716 (48.2%)	
Female	3247 (51.4%)	271 (49.6%)	1128 (50.9%)	1848 (51.8%)	
Race					**< 0.001**
Mexican American	704 (7.8%)	23 (2.5%)	250 (8.4%)	431 (8.1%)	
Other Hispanic	582 (5.4%)	43 (4.3%)	250 (7.8%)	289 (4.5%)	
Non‐Hispanic white	2710 (68.4%)	274 (71.7%)	766 (58.6%)	1670 (72.3%)	
Non‐Hispanic Black	1436 (10.7%)	163 (13.9%)	712 (18.3%)	561 (7.1%)	
Non‐Hispanic Asian	689 (5.2%)	26 (2.1%)	141 (4.1%)	522 (6.0%)	
Other race‐including multi‐racial	170 (2.5%)	24 (5.4%)	55 (3.0%)	91 (2.0%)	
Education level					**< 0.001**
Less than 9th grade	512 (5.0%)	92 (11.7%)	253 (8.0%)	167 (3.0%)	
9–11th grade	837 (9.9%)	153 (25.4%)	389 (15.6%)	295 (5.9%)	
High school graduate/GED or equivalent	1386 (20.9%)	155 (30.6%)	629 (31.8%)	602 (15.2%)	
Some college or AA degree	1807 (30.1%)	117 (24.8%)	619 (30.9%)	1071 (30.3%)	
College graduate or above	1746 (34.2%)	36 (7.4%)	281 (13.7%)	1429 (45.6%)	
Don't know	3 (0.0%)	0 (0.0%)	3 (0.0%)	0 (0.0%)	
Income‐poverty ratio	2.9 (1.3–5.0)	1.6 (0.9–2.7)	1.8 (0.9–3.2)	3.8 (1.8–5.0)	**< 0.001**
BMI (kg/m^2^)	28.0 (24.6–32.5)	28.4 (24.5–33.0)	28.8 (25.1–33.8)	27.6 (24.4–31.6)	**< 0.001**
Energy intake (kcal)	1973.5 (1563.0–2492.0)	1729.0 (1318.0–2288.5)	1949.5 (1496.0–2430.0)	2008.5 (1610.5–2543.5)	**< 0.001**
nif‐Tooth count	26.0 (22.0–28.0)	0.0 (0.0–0.0)	20.0 (14.0–23.0)	28.0 (26.0–28.0)	**< 0.001**
nif‐FTU	10.0 (4.0–12.0)	0.0 (0.0–0.0)	3.0 (0.0–6.0)	12.0 (10.0–12.0)	**< 0.001**
Alcohol consumption					**< 0.001**
Everyday	260 (5.2%)	18 (2.3%)	107 (6.5%)	135 (4.9%)	
Every week	1619 (30.8%)	75 (14.4%)	423 (21.8%)	1121 (36.3%)	
Every month/year	959 (17.0%)	35 (5.6%)	261 (12.1%)	663 (20.2%)	
Several month/year	1178 (17.0%)	111 (22.3%)	440 (19.3%)	627 (15.5%)	
Never	1152 (15.2%)	212 (35.4%)	516 (22.8%)	424 (9.9%)	
Missing	1123 (14.9%)	102 (19.9%)	427 (17.5%)	594 (13.2%)	
Smoking status					**< 0.001**
Never	3450 (55.0%)	169 (30.1%)	990 (41.8%)	2291 (63.2%)	
Former smoker	1667 (26.9%)	212 (38.1%)	628 (28.4%)	827 (25.1%)	
Current smoker	1170 (18.1%)	172 (31.8%)	554 (29.8%)	444 (11.7%)	
Missing	4 (0.0%)	0 (0.0%)	2 (0.0%)	2 (0.0%)	
Blood pressure status					**< 0.001**
Normal	2539 (42.7%)	159 (31.8%)	683 (34.9%)	1697 (47.1%)	
Elevated	1064 (17.5%)	89 (16.9%)	376 (17.4%)	599 (17.6%)	
Hypertension stage 1	1349 (21.4%)	118 (21.4%)	525 (21.6%)	706 (21.4%)	
Hypertension stage 2	1168 (16.1%)	162 (25.8%)	507 (22.7%)	499 (12.4%)	
Hypertension Crisis	45 (0.4%)	11 (0.9%)	30 (1.2%)	4 (0.0%)	
Missing	126 (1.8%)	14 (3.2%)	53 (2.3%)	59 (1.5%)	
Diabetes status					**< 0.001**
Non‐diabetes	3384 (61.8%)	199 (44.4%)	939 (48.1%)	2246 (69.5%)	
Prediabetes	1940 (27.2%)	218 (36.3%)	787 (35.8%)	935 (22.6%)	
Diabetes	512 (5.5%)	72 (11.6%)	252 (8.4%)	188 (3.6%)	
Uncontrolled diabetes	280 (3.2%)	44 (5.8%)	132 (5.3%)	104 (2.0%)	
Missing	175 (2.3%)	20 (1.9%)	64 (2.3%)	91 (2.3%)	

*Note:* nif represent the consideration including natural tooth, dental implant and fixed restorations for tooth count or functional tooth unit. BMI (body mass index): calculated as weight (kg) divided by height squared (m^2^). Income‐poverty ratio: calculated by dividing total family income by the poverty threshold based on poverty guidelines, specific to family size, year and state. Smoking status: non‐smoker, smoked < 100 cigarettes in life; former smoker, smoked ≥ 100 cigarettes in life and quit smoking; and current smoker, smoked ≥ 100 cigarettes in life and currently smoking. Blood pressure status: categorised based on examined systolic and diastolic blood pressure values (SBP and DBP); Normal, SBP < 120 mmHg and DBP < 80 mmHg; Elevated, SBP 120–129 mmHg and DBP < 80 mmHg; hypertension stage I, SBP 130–139 mmHg or DBP 80–89 mmHg; hypertension stage II, SBP ≥ 140 mmHg or DBP ≥ 90 mmHg; hypertension crisis, SBP > 180 mmHg and/or DBP > 120 mmHg. Diabetes status: defined by tested glycohaemoglobin (HbA1c) levels. Non‐diabetes, HbA1c < 5.7%; Prediabetes, HbA1c 5.7%–6.4%; Diabetes, HbA1c ≥ 6.5%; poorly controlled diabetes, HbA1c ≥ 8.0%. All estimates accounted for the NHANES complex sampling design, including sampling weights, strata and primary sampling units. Categorical variables are presented as unweighted counts and survey‐weighted percentages. Continuous variables are presented as survey‐weighted medians and interquartile ranges (IQRs). *p*‐values for categorical variables were calculated using survey‐weighted chi‐square tests, and *p*‐values for continuous variables were calculated using survey‐weighted Kruskal–Wallis rank tests. Significant statistical differences of *p* < 0.05 was labled in bold.

Abbreviations: Adequate FTUs, FTU ≥ 8; FTU, functional tooth units; Impaired FTUs, FTU < 8.

### 3.2 Associations Between FTUs and Dietary Quality

**FIGURE 1:**
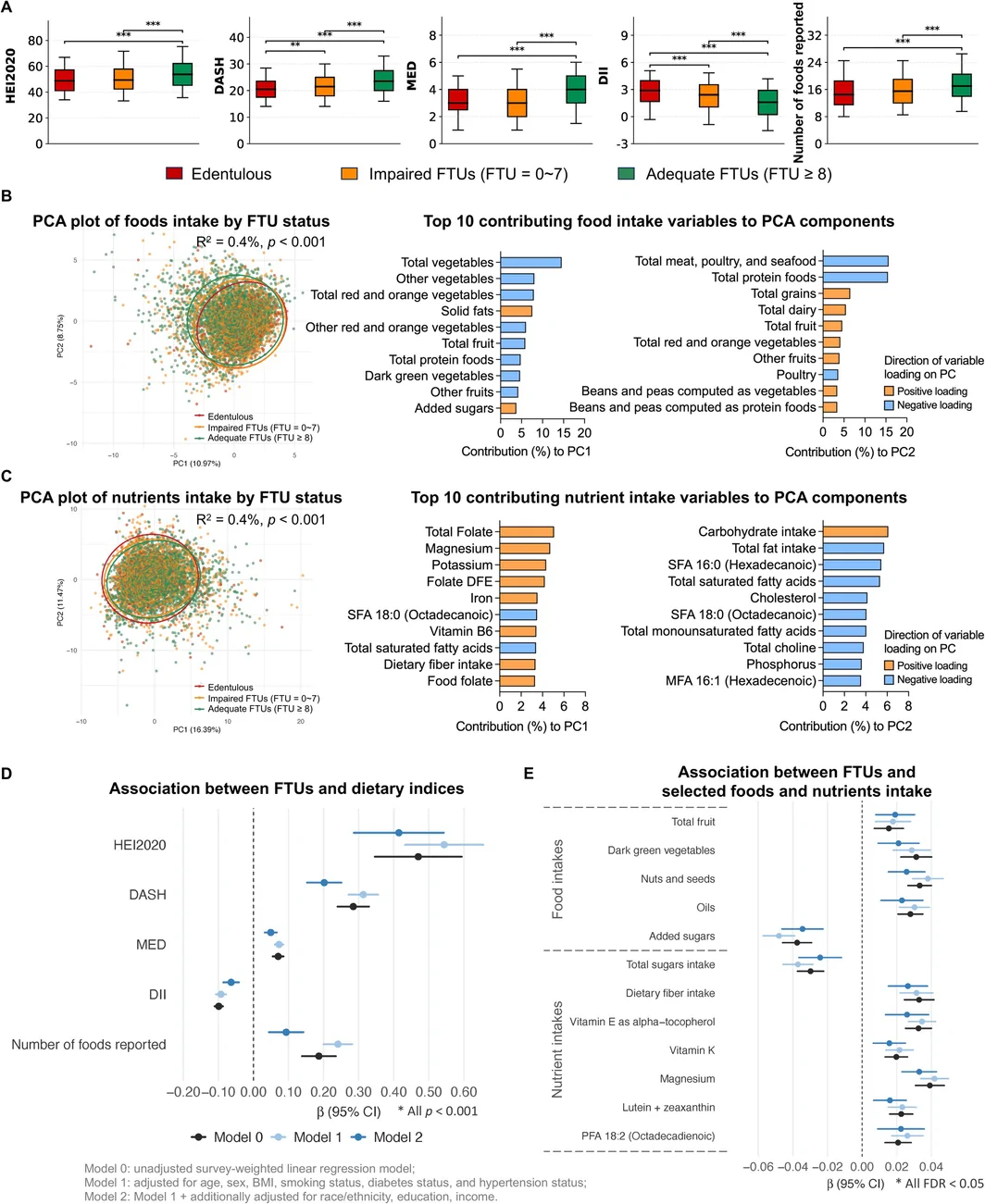
Associations between functional tooth units and dietary intake patterns. (A) Comparison of dietary indices and the number of foods reported across functional tooth unit (FTU) categories. (B) Principal component analysis (PCA) of food intake variables by FTU category, with bar plots showing the top 10 food intake variables contributing to PC1 and PC2. (C) PCA of nutrient intake variables by FTU category, with bar plots showing the top 10 nutrient intake variables contributing to PC1 and PC2. (D) Forest plot showing the associations of FTUs with dietary indices and the number of foods reported. (E) Forest plot showing the associations of FTUs with selected food and nutrient intake variables. All analyses accounted for the NHANES complex survey design. In (A), variables are presented as survey‐weighted medians and interquartile ranges. Overall group differences were tested using survey‐weighted Kruskal–Wallis rank tests, followed by survey‐weighted Wilcoxon rank‐sum tests with Bonferroni correction for post hoc pairwise comparisons. Statistical significance is indicated as *p < 0.05, **p < 0.01, ***p < 0.001. For the PCA plots in (B) and (C), PCA was performed using food or nutrient intake variables that were energy‐adjusted per 1000 kcal and *z*‐score‐standardised. Each point represents one participant, and ellipses indicate 95% confidence regions. Permutational multivariate analysis of variance (PERMANOVA) was used to estimate the explained variance (*R* ^2^) of FTU categories in the PCA space. Explained variance refers to the proportion of statistical variation in PCA‐derived dietary profiles associated with each factor in PERMANOVA and should not be interpreted causally. The food and nutrient variables included in the PCA are listed in Table S2. For the loading plots in (B) and (C), bar length indicates the percentage contribution of each variable to the corresponding principal component. Bar colour indicates the loading direction on that component: Blue indicates negative loadings, and yellow indicates positive loadings. Therefore, higher intake of a variable with a negative or positive loading contributes to a shift towards lower or higher values of the corresponding principal component, respectively. For (D) and (E), associations were estimated using survey‐weighted linear regression models. nif‐FTUs was specified as exposures, and variables of dietary intakes were specified as outcomes for statistical modelling and presentation. Model 0 was unadjusted; Model 1 was adjusted for age, sex, BMI, smoking status, diabetes status and hypertension status; and Model 2 was additionally adjusted for race/ethnicity, education level and income‐poverty ratio. Beta estimates and 95% confidence intervals are shown as points and error bars. Associations were considered statistically significant when the 95% confidence interval did not cross the dashed line at beta = 0. In (E), selected food and nutrient intake variables were defined as variables significantly associated with FTUs in the fully adjusted model (Model 2), with Benjamini–Hochberg false discovery rate (FDR) correction < 0.05. All food and nutrient intake variables were energy‐adjusted using the per 1000 kcal method and *z*‐score‐standardised. FTU, functional tooth unit; nif, FTU definition considering natural teeth, dental implants and fixed restorations; HEI2020, Healthy Eating Index 2020; DASH, Dietary Approaches to Stop Hypertension; MED, Mediterranean Diet Score; DII, Dietary Inflammatory Index.

Dietary quality differed across FTU categories (Figure 1A, Table S2). Participants with adequate FTUs had more favourable dietary profiles than edentulous participants and those with impaired FTUs, reflected by higher HEI‐2020, DASH and MED scores, lower DII scores and a larger number of reported food items. Median HEI‐2020 increased from 48.66 in the edentulous group and 49.36 in the impaired‐FTU group to 53.75 in the adequate‐FTU group, while median DII decreased from 2.88 and 2.42 to 1.59, respectively. Participants with impaired FTUs also showed more favourable DASH and DII scores than edentulous participants. PCA suggested statistically significant, although limited, differences in the overall food group and nutrient intake profiles across FTU categories (*R* ^2^ = 0.4% for both; Figure 1B,C). The top contributing variables were further identified. For food group intake, total vegetables, total meat, poultry and seafood and total protein foods were among the strongest contributors to between‐group variation. For nutrient intake, the contributions were more evenly distributed across principal components.

We next examined the relationship between the number of FTUs and dietary quality. In both crude and adjusted models, a higher number of FTUs was consistently associated with more favourable dietary pattern indices and a greater number of reported food items (Figure 1D). In the fully adjusted model, each additional FTU was associated with higher HEI‐2020 (β = 0.41, 95% CI: 0.29–0.54), DASH (β = 0.20, 95% CI: 0.15–0.25) and MED scores (β = 0.05, 95% CI: 0.03–0.07), lower DII scores (β = −0.06, 95% CI: −0.09 to −0.04) and a greater number of reported food items (β = 0.09, 95% CI: 0.04–0.14). Higher FTUs were also associated with many food and nutrient intakes, including greater consumption of fruit, dark green vegetables, nuts and seeds, oils, dietary fibre, selected micronutrients and polyunsaturated fatty acid 18:2, and lower intake of added or total sugars (Figure 1E). Sensitivity analysed based on tooth count showed patterns generally consistent with the FTU‐based analyses (Figure S2, Table S3).

### 3.3 Associations of Oral Disease Status and Disease Extent With Dietary Quality

Dietary quality differences across oral disease status among dentate participants were further assessed, with and without adjustment for FTUs. Among the 5738 dentate participants included in this analysis, 2455 had no untreated caries or periodontitis, 561 had untreated caries only, 1731 had periodontitis only and 991 had coexisting untreated caries and periodontitis (Table 3). All sociodemographic and health‐related characteristics differed significantly across oral disease subgroups, except for total energy intake.

**TABLE 3:** Characteristics of 5738 dentate individuals classified by oral conditions considering untreated caries and periodontitis.

Variables	All dentate individuals (*n* = 5738)	No untreated caries/periodontitis (*n* = 2455)	Untreated caries only (*n* = 561)	Periodontitis only (*n* = 1731)	Untreated caries + periodontitis (*n* = 991)	*p* value
Age (year)	50.0 (40.0–61.0)	48.0 (39.0–59.0)	41.0 (36.0–52.0)	58.0 (46.0–66.0)	51.0 (40.0–59.0)	**< 0.001**
Sex						**< 0.001**
Male	2762 (48.4%)	978 (42.7%)	240 (44.5%)	958 (55.6%)	586 (58.6%)	
Female	2976 (51.6%)	1477 (57.3%)	321 (55.5%)	773 (44.4%)	405 (41.4%)	
Race						**< 0.001**
Mexican American	681 (8.2%)	204 (5.6%)	71 (9.6%)	232 (9.2%)	174 (15.0%)	
Other Hispanic	539 (5.5%)	218 (4.7%)	45 (5.9%)	200 (6.8%)	76 (5.7%)	
Non‐Hispanic White	2436 (68.2%)	1246 (75.4%)	235 (60.8%)	614 (63.6%)	341 (54.1%)	
Non‐Hispanic Black	1273 (10.5%)	386 (6.5%)	137 (16.3%)	431 (12.2%)	319 (18.4%)	
Non‐Hispanic Asian	663 (5.4%)	336 (5.6%)	51 (4.2%)	221 (6.4%)	55 (3.4%)	
Other race‐including multi‐racial	146 (2.3%)	65 (2.2%)	22 (3.2%)	33 (1.8%)	26 (3.3%)	
Education level						**< 0.001**
Less than 9th grade	420 (4.5%)	91 (2.3%)	32 (4.5%)	168 (5.3%)	129 (11.0%)	
9–11th grade	684 (8.8%)	169 (4.6%)	74 (10.7%)	228 (10.5%)	213 (19.8%)	
High school graduate/GED or equivalent	1231 (20.2%)	372 (13.2%)	133 (25.8%)	428 (25.2%)	298 (33.1%)	
Some college or AA degree	1690 (30.5%)	731 (30.1%)	207 (39.1%)	504 (30.6%)	248 (26.6%)	
College graduate or above	1710 (36.1%)	1092 (49.7%)	114 (19.8%)	402 (28.4%)	102 (9.4%)	
Don't know	3 (0.0%)	0 (0.0%)	1 (0.1%)	1 (0.0%)	1 (0.0%)	
Income‐poverty ratio	3.1 (1.3–5.0)	4.2 (2.1–5.0)	1.8 (0.9–3.9)	2.6 (1.3–4.8)	1.5 (0.9–2.8)	**< 0.001**
BMI (kg/m^2^)	28.0 (24.6–32.4)	27.5 (24.3–31.4)	29.7 (25.6–34.0)	28.0 (24.8–32.6)	28.8 (25.1–34.5)	**< 0.001**
Energy intake (kcal)	1983.5 (1580.5–2503.5)	1973.0 (1580.5–2450.5)	2052.5 (1648.0–2612.5)	2001.0 (1600.0–2516.5)	2023.0 (1490.0–2628.0)	0.282
Decay %	0.0 (0.0–0.0)	0.0 (0.0–0.0)	1.9 (0.8–3.4)	0.0 (0.0–0.0)	3.2 (1.4–7.0)	**< 0.001**
Decay + fillings %	14.5 (5.8–27.9)	14.3 (5.7–27.1)	14.3 (7.1–24.5)	17.1 (6.2–33.1)	11.5 (5.7–22.5)	**0.002**
PD ≥ 4 mm %	0.0 (0.0–1.2)	0.0 (0.0–0.0)	0.0 (0.0–0.0)	1.6 (0.0–4.9)	3.2 (0.7–9.6)	**< 0.001**
CAL ≥ 1 mm %	92.3 (82.0–98.4)	85.8 (75.7–93.5)	90.1 (79.2–95.2)	98.0 (93.0–100.0)	99.2 (95.2–100.0)	**< 0.001**
CAL ≥ 3 mm %	7.7 (1.8–23.8)	2.4 (0.6–6.4)	5.6 (1.8–9.4)	25.0 (14.5–44.7)	39.9 (22.5–65.4)	**< 0.001**
nif‐Tooth count	27.0 (24.0–28.0)	28.0 (26.0–28.0)	25.0 (22.0–28.0)	26.0 (21.0–28.0)	22.0 (17.0–26.0)	**< 0.001**
Tooth count category						**< 0.001**
nif‐Tooth count = 1–19	1052 (13.5%)	171 (3.9%)	105 (18.4%)	424 (19.3%)	352 (35.3%)	
nif‐Tooth count ≥ 20	4686 (86.5%)	2284 (96.1%)	456 (81.6%)	1307 (80.7%)	639 (64.7%)	
nif‐FTU	10.0 (6.0–12.0)	12.0 (10.0–12.0)	8.0 (4.0–12.0)	9.0 (3.0–12.0)	4.0 (1.0–9.0)	**< 0.001**
FTU category						**< 0.001**
nif‐FTU = 0–7	2174 (30.0%)	458 (12.2%)	248 (44.7%)	796 (40.7%)	672 (67.1%)	
nif‐FTU ≥ 8	3564 (70.0%)	1997 (87.8%)	313 (55.3%)	935 (59.3%)	319 (32.9%)	
Alcohol consumption						**< 0.001**
Everyday	242 (5.4%)	78 (4.3%)	15 (3.4%)	88 (7.7%)	61 (6.5%)	
Every week	1544 (32.0%)	735 (36.2%)	140 (27.4%)	445 (29.9%)	224 (22.8%)	
Every month/year	924 (17.8%)	463 (20.0%)	97 (19.5%)	242 (14.5%)	122 (14.4%)	
Several month/year	1067 (16.6%)	448 (15.5%)	116 (19.1%)	312 (17.9%)	191 (16.9%)	
Never	940 (13.7%)	298 (9.4%)	93 (14.2%)	333 (17.7%)	216 (22.0%)	
Missing	1021 (14.5%)	433 (14.5%)	100 (16.5%)	311 (12.4%)	177 (17.3%)	
Smoking status						**< 0.001**
Never	3281 (56.8%)	1672 (67.0%)	306 (49.0%)	896 (48.5%)	407 (38.7%)	
Former smoker	1455 (26.1%)	579 (25.6%)	112 (20.5%)	523 (31.1%)	241 (22.0%)	
Current smoker	998 (17.1%)	204 (7.4%)	143 (30.4%)	310 (20.3%)	341 (39.2%)	
Missing	4 (0.0%)	0 (0.0%)	0 (0.0%)	2 (0.1%)	2 (0.1%)	
Blood pressure status						**< 0.001**
Normal	2380 (43.4%)	1207 (48.7%)	249 (44.9%)	579 (36.3%)	345 (36.4%)	
Elevated	975 (17.5%)	388 (16.5%)	94 (18.9%)	334 (18.4%)	159 (19.2%)	
Hypertension stage 1	1231 (21.4%)	489 (21.6%)	113 (17.8%)	401 (21.3%)	228 (23.1%)	
Hypertension stage 2	1006 (15.4%)	330 (11.7%)	86 (16.0%)	366 (21.3%)	224 (18.1%)	
Hypertension Crisis	34 (0.4%)	7 (0.2%)	3 (0.3%)	15 (0.9%)	9 (0.4%)	
Missing	112 (1.7%)	34 (1.4%)	16 (2.1%)	36 (1.8%)	26 (2.8%)	
Diabetes status						**< 0.001**
Non‐diabetes	3185 (63.1%)	1572 (72.7%)	345 (66.2%)	780 (47.8%)	488 (54.2%)	
Prediabetes	1722 (26.6%)	633 (20.6%)	145 (24.9%)	637 (37.2%)	307 (29.7%)	
Diabetes	440 (5.1%)	151 (3.7%)	36 (4.0%)	155 (6.8%)	98 (7.3%)	
Uncontrolled diabetes	236 (3.0%)	44 (1.2%)	16 (1.7%)	104 (5.1%)	72 (6.7%)	
Missing	155 (2.3%)	55 (1.8%)	19 (3.2%)	55 (3.1%)	26 (2.0%)	

*Note:* nif represent the consideration including natural tooth, dental implant and fixed restorations for tooth count or functional tooth unit. BMI (body mass index): calculated as weight (kg) divided by height squared (m^2^). Income‐poverty ratio: calculated by dividing total family income by the poverty threshold based on poverty guidelines, specific to family size, year and state. Decay% represents the proportion of assessed tooth surfaces with untreated caries. Decay + Filling% represents the proportion of assessed tooth surfaces with either untreated caries or restorations. Assessed tooth surfaces included occlusal, facial, lingual, mesial and distal surfaces. PD, probing depth; PD ≥ 4 mm% represents the proportion of assessed periodontal sites with PD ≥ 4 mm. CAL, clinical attachment loss; CAL ≥ 1 mm% and CAL ≥ 3 mm% represent the proportions of assessed periodontal sites with CAL ≥ 1 mm and ≥ 3 mm, respectively. Assessed periodontal sites included mesial, mid and distal sites on both buccal and lingual surfaces. Smoking status: non‐smoker, smoked < 100 cigarettes in life; former smoker, smoked ≥ 100 cigarettes in life and quitted from smoking; and current smoker, smoked ≥ 100 cigarettes in life and currently smoking. Blood pressure status: categorised based on examined systolic and diastolic blood pressure values (SBP and DBP); Normal, SBP < 120 mmHg and DBP < 80 mmHg; Elevated, SBP 120–129 mmHg and DBP < 80 mmHg; hypertension stage I, SBP 130–139 mmHg or DBP 80–89 mmHg; hypertension stage II, SBP ≥ 140 mmHg or DBP ≥ 90 mmHg; hypertension crisis, SBP > 180 mmHg and/or DBP > 120 mmHg. Diabetes status: defined by tested glycohaemoglobin (HbA1c) levels. Non‐diabetes, HbA1c < 5.7%; Prediabetes, HbA1c 5.7%–6.4%; Diabetes, HbA1c ≥ 6.5%; Poorly controlled diabetes, HbA1c ≥ 8.0%. All estimates accounted for the NHANES complex sampling design, including sampling weights, strata, and primary sampling units. Categorical variables are presented as unweighted counts and survey‐weighted percentages. Continuous variables are presented as survey‐weighted medians and interquartile ranges (IQRs). *p*‐values for categorical variables were calculated using survey‐weighted chi‐square tests, and *p* values for continuous variables were calculated using survey‐weighted Kruskal–Wallis rank tests. Significant statistical differences of *p* < 0.05 was labled in bold.

Abbreviations: Adequate FTUs, FTU ≥ 8; FTU, functional tooth units; Impaired FTUs, FTU < 8.

**FIGURE 2:**
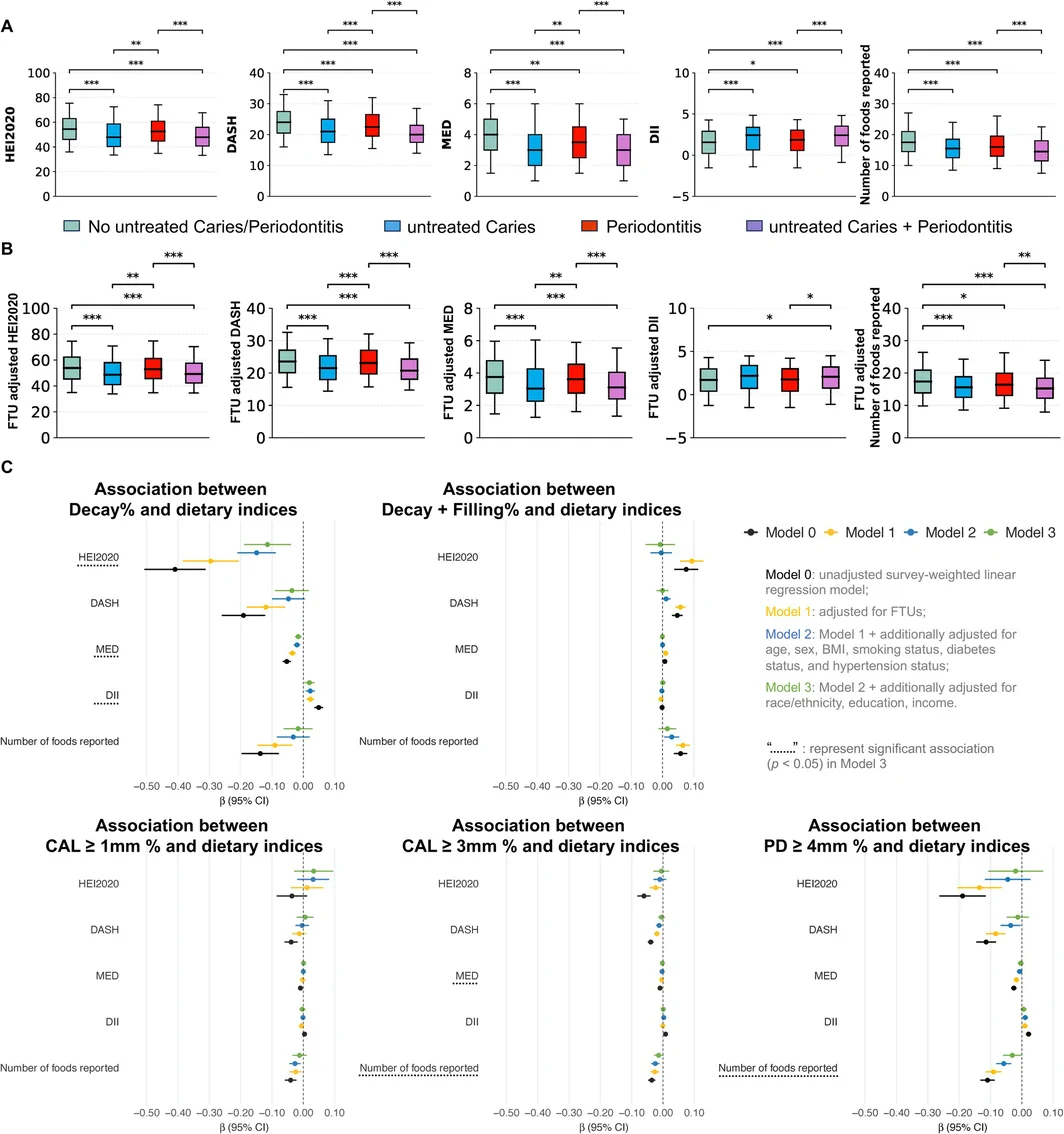
Associations of oral disease burden with dietary indices and the number of foods reported in dentate individuals. (A) Comparison of dietary indices and the number of foods reported across oral disease conditions. (B) Comparison of dietary indices and the number of foods reported across oral disease conditions, after adjustment for nif‐FTUs. (C) Forest plots showing the associations of oral disease extent, including Decay%, Decay + Filling%, CAL ≥ 1 mm%, CAL ≥ 3 mm% and PD ≥ 4 mm%, with dietary indices and the number of foods reported. All analyses accounted for the NHANES complex survey design. Oral disease conditions were defined according to untreated caries and periodontitis status, resulting in four groups: no untreated caries or periodontitis; untreated caries only; periodontitis only; and co‐existing untreated caries and periodontitis. In (A) and (B), variables are presented as survey‐weighted medians and interquartile ranges. Overall group differences were tested using survey‐weighted Kruskal–Wallis rank tests, followed by survey‐weighted Wilcoxon rank‐sum tests with Bonferroni correction for post hoc pairwise comparisons. Statistical significance is indicated as *p < 0.05, **p < 0.01, ***p < 0.001. In (B), dietary indices and the number of foods reported were adjusted for nif‐FTUs using a survey‐weighted residual adjustment approach. Adjusted values were derived by removing the variation explained by nif‐FTUs while preserving the original survey‐weighted mean of each dietary variable. For (C), associations were estimated using survey‐weighted linear regression models. Variables of oral disease extent were specified as exposures and variables of dietary indices and the number of foods reported were specified as outcomes for statistical modelling and presentation. Model 0 was unadjusted; Model 1 was adjusted for nif‐FTUs; Model 2 was additionally adjusted for age, sex, BMI, smoking status, diabetes status and hypertension status; and Model 3 was further adjusted for race/ethnicity, education level and income‐poverty ratio. Beta estimates and 95% confidence intervals are shown as points and error bars. Associations were considered statistically significant when the 95% confidence interval did not cross the dashed line at beta = 0. Dashed underline highlights associations that remained significant in the fully adjusted model (Model 3). FTU, functional tooth unit; nif, FTU definition considering natural teeth, dental implants and fixed restorations; HEI2020, Healthy Eating Index 2020; DASH, Dietary Approaches to Stop Hypertension; MED, Mediterranean Diet Score; DII, Dietary Inflammatory Index; CAL, clinical attachment loss; PD, probing depth.

Across the four oral disease subgroups, significant differences were observed in all dietary indices, in the number of reported food items (Figure 2A) and in multiple food and nutrient variables (Table S4). Participants without caries or periodontitis generally showed the most favourable dietary profiles, whereas those with untreated caries, either alone or coexisting with periodontitis, tended to have lower HEI‐2020, DASH and MED scores, higher DII scores and fewer reported food items.

After FTU adjustment, differences for the periodontitis‐only group were attenuated, whereas untreated‐caries‐related groups continued to show less favourable dietary profiles (Figure 2B).

**FIGURE 3:**
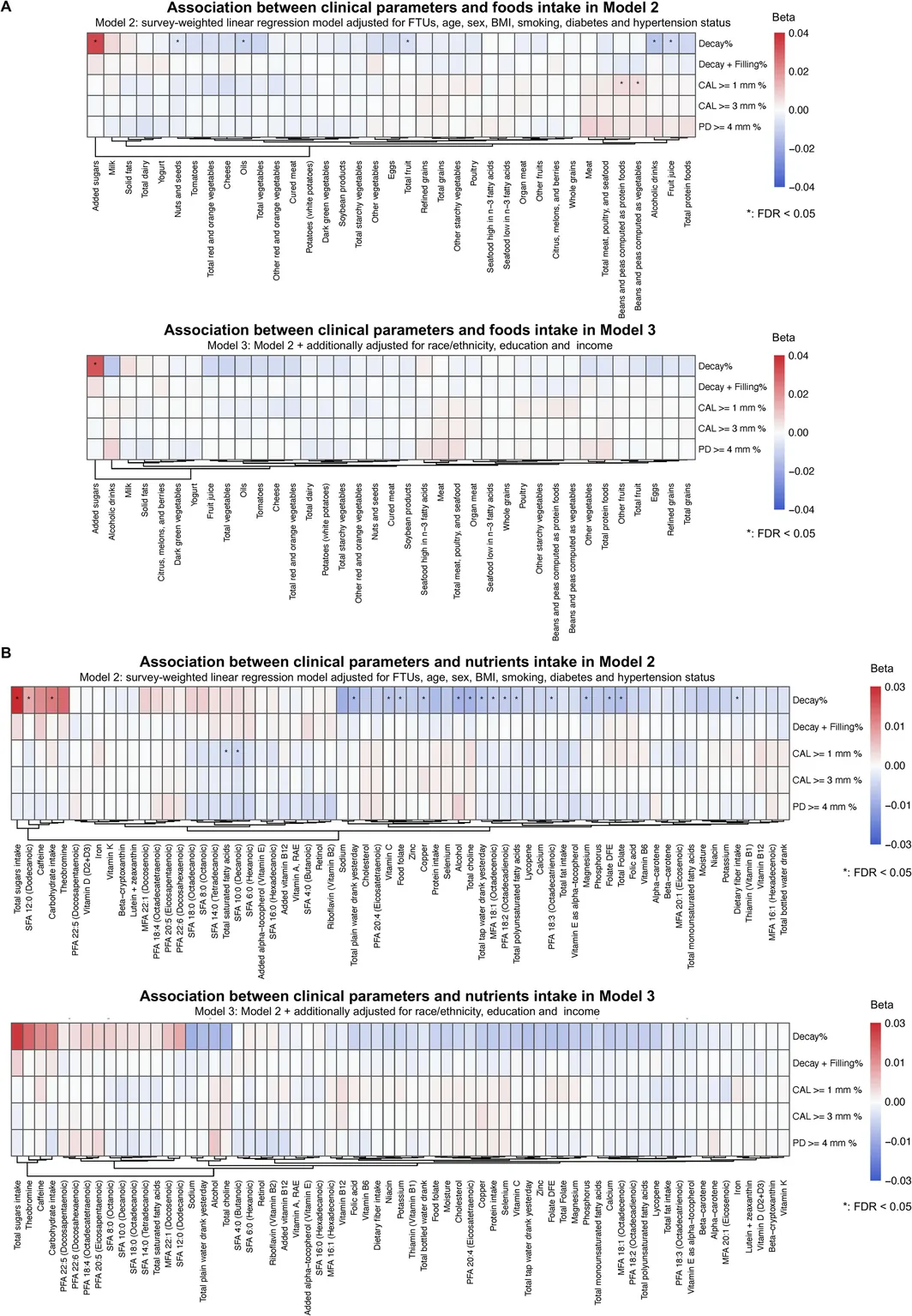
Associations of oral disease extent with food and nutrient intake. (A) Clustered heatmaps showing the associations of oral disease extent, including Decay%, Decay + Filling%, CAL ≥ 1 mm%, CAL ≥ 3 mm% and PD ≥ 4 mm%, with food intake variables in different adjustment models. (B) Clustered heatmaps showing the associations of oral disease extent with nutrient intake variables in different adjustment models. All analyses accounted for the NHANES complex survey design. Associations were estimated using survey‐weighted linear regression models. Variables of oral disease extent were specified as exposures and variables of food and nutrient intakes as outcomes for statistical modelling and presentation. All food and nutrient intake variables were energy‐adjusted using the per 1000 kcal method and *z*‐score‐standardised. Model 2 and 3 shown in this figure were the same as those used in Figure 2C. Model 2 was adjusted for nif‐FTUs, age, sex, BMI, smoking status, diabetes status and hypertension status; and Model 3 was additionally adjusted for race/ethnicity, education level and income‐poverty ratio. Benjamini–Hochberg false discovery rate (FDR) correction was applied for multiple comparison, and statistical significance is indicated by *FDR < 0.05. FTU, functional tooth unit; nif, FTU definition considering natural teeth, dental implants, and fixed restorations; CAL, clinical attachment loss; PD, probing depth. Corresponding results of unadjusted model (Model 0) and model adjusted for nif‐FTUs (Model 1) were provided in Figure S3.

In linear regression, both caries‐ and periodontitis‐related extent parameters were associated with most dietary indices in crude and FTU‐adjusted models (Figure 2C). After full adjustment, associations were more consistently retained for untreated caries: higher Decay% remained significantly associated with lower HEI‐2020, MED scores and higher DII scores, whereas Decay + Filling% showed no significant associations. For periodontal parameters, PD ≥ 4 mm and CAL ≥ 3 mm were significantly associated with fewer reported food items, and CAL ≥ 3 mm was also associated with lower MED scores in fully adjusted models. Associations between disease extent and specific food groups or nutrient intakes are shown in Figure 3 and Figure S3. In Model 2, significant associations were mainly observed for Decay% and CAL ≥ 1 mm%. After further full adjustment for socioeconomic factors, only the positive association between Decay% and added sugar intake remained significant.

### 3.4 Variance in Dietary Profiles Explained by Oral and Non‐Oral Factors

**FIGURE 4:**
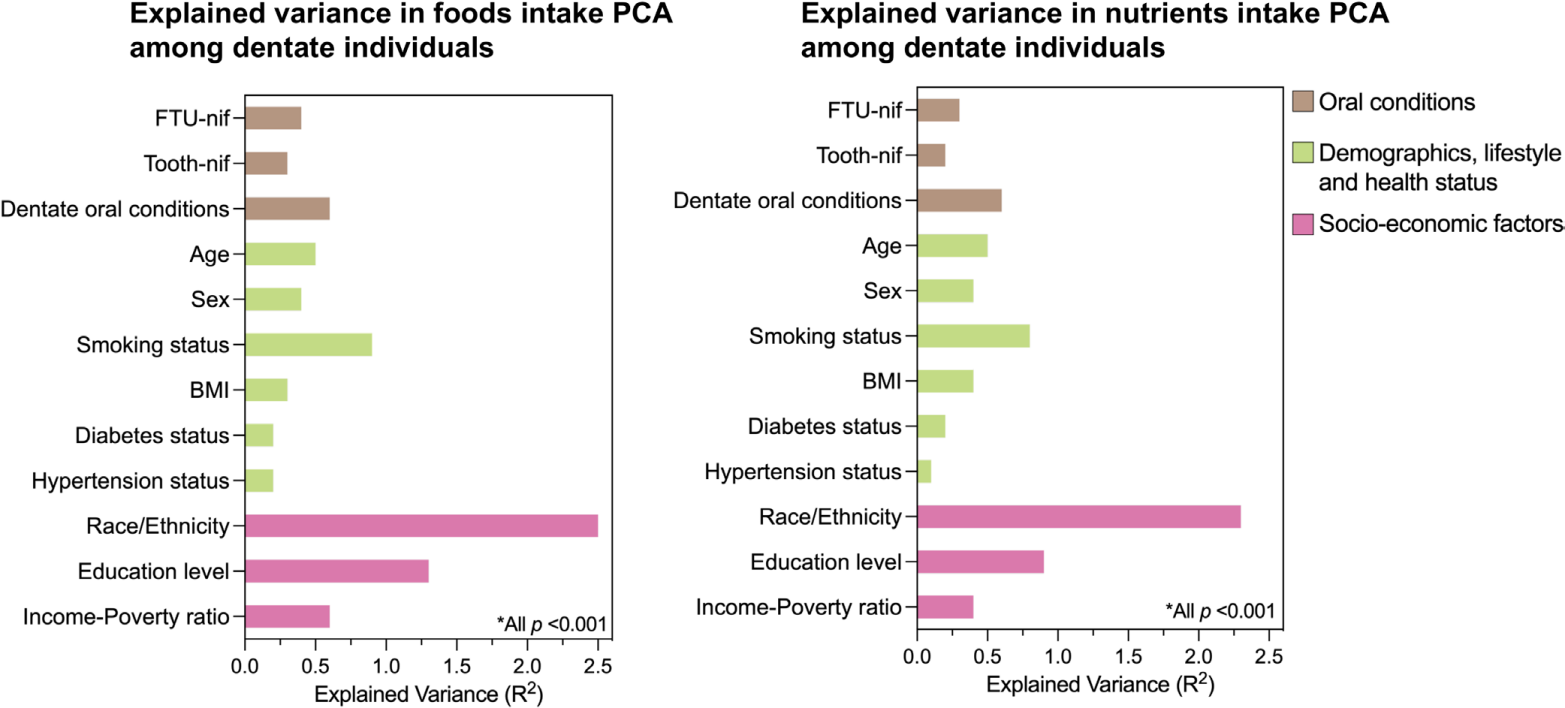
Variance in overall food and nutrient intake explained by oral conditions and participant characteristics in dentate individuals. The variance in overall food or nutrient intake explained by oral conditions, demographic factors, lifestyle factors, health status and socioeconomic factors was estimated using permutational multivariate analysis of variance (PERMANOVA) based on principal component analysis (PCA) of food and nutrient intake variables. Explained variance refers to the proportion of statistical variation in PCA‐derived dietary profiles associated with each factor in PERMANOVA and should not be interpreted causally. PCA was performed using food or nutrient intake variables that were energy‐adjusted per 1000 kcal and *z*‐score‐standardised. Dentate oral conditions were defined according to untreated caries and periodontitis status, resulting in four groups of no untreated caries or periodontitis, untreated caries only, periodontitis only and co‐existing untreated caries and periodontitis. The food and nutrient variables included in the PCA are listed in Table S2. FTU‐nif, functional tooth unit considering natural teeth, dental implants and fixed restorations; Tooth‐nif, tooth numbers considering natural teeth, dental implants and fixed restorations.

To place the above associations in a broader context, we assessed the variance in PCA‐derived food and nutrient intake profiles explained by oral and non‐oral factors among dentate participants (Figure 4). FTUs, tooth count and oral disease status were all significantly associated with variances in overall food or nutrient intake profiles. Among the oral factors, oral disease status showed the largest explained variance for both food intake and nutrient intake profiles (*R* ^2^ = 0.6% for both), exceeding that of FTUs (*R* ^2^ = 0.4% and 0.3%, respectively) and tooth count (*R* ^2^ = 0.3% and 0.2%, respectively). However, the largest explained variance was observed for sociodemographic factors, particularly race/ethnicity (*R* ^2^ = 2.5% for food intake and 2.3% for nutrient intake) and education level (*R* ^2^ = 1.3% and 0.9%, respectively). Similar patterns were observed when both dentate and edentulous participants were included in the analysis (Figure S4).

## 4 Discussion

This study evaluated associations between dentition status, oral diseases and dietary quality in a nationally representative sample of US adults. Four findings emerged. First, higher FTU counts were associated with better dietary quality. Second, dietary quality differed by oral disease status, with less favourable profiles most consistently observed in untreated caries‐related subgroups, even after accounting for FTUs. Third, the extent of untreated decay showed more consistent associations with dietary indices than Decay + Filling% or the periodontal parameters available in NHANES. Finally, although oral conditions were significantly associated with overall dietary quality, their explained variance on overall food or nutrient intakes was smaller than that of sociodemographic factors.

Our findings extend previous work that primarily examined tooth loss, edentulism or prosthetic rehabilitation as proxies for masticatory function (Gaewkhiew et al. 2019; Sheiham and Steele 2001) while acknowledging that FTUs do not fully capture masticatory function. The consistent associations of both FTU category and FTU count with dietary indices, food diversity and selected food and nutrient intakes suggest that posterior occlusal support is an important oral‐health correlate of dietary profiles.

Furthermore, prior investigations have examined diet in relation to either periodontitis or dental caries separately, despite their frequent coexistence and potential shared aetiology. For periodontitis, several studies have shown that less favourable or more pro‐inflammatory dietary patterns are associated with greater prevalence or severity (A. Li et al. 2021; Sheiham and Steele 2001; Shi et al. 2025), whereas dental caries has been consistently linked to cariogenic behaviours, particularly higher free‐sugar intake and frequent consumption of refined carbohydrates (Mazurkiewicz et al. 2023; van Meijeren‐van Lunteren et al. 2023). Only a few studies have simultaneously assessed dietary factors for both diseases, and even there caries and periodontitis were typically modelled separately (Yoshihara et al. 2009). Broader consensus reports have emphasised that while both diseases share common lifestyle‐related determinants, their dietary pathways differ: caries being sugar‐driven and periodontitis more related to systemic inflammatory and metabolic profiles (Chapple et al. 2017; Hujoel and Lingström 2017).

In contrast, the present study incorporated three oral health indicators—FTUs, untreated caries and periodontitis—into a single analytical framework. This approach allowed us to examine FTUs, untreated caries and periodontitis within the same analytical framework and to evaluate how their associations with dietary quality changed after covariate adjustment. Such an approach may also help identify distinct oral health subgroups associated with adverse dietary profiles, thereby helping to identify subgroups that may warrant closer dietary assessment or integrated oral–nutritional care.

In the current framework, untreated caries‐related groups continued to show less favourable dietary profiles even after adjusting for FTUs, while differences for the periodontitis‐only group were attenuated. This suggests that the observed periodontitis–diet association may be closely linked to reduced FTUs. In addition, the clinical extent of periodontitis showed generally weaker associations with dietary quality, which may reflect that the parameters used (PD, CAL) do not fully capture dimensions of periodontal disease most directly relevant to eating behaviour, such as symptoms, tooth mobility or functional instability. However, these findings do not imply that caries is intrinsically more relevant to diet than periodontitis; rather, within the present operational definitions and analytical framework, caries‐related subgroups exhibited more consistent associations with dietary quality than the available periodontal parameters.

The sugar‐related findings warrant particular attention. In the analyses of oral disease extent, the positive association between Decay% and added sugar intake was the only food‐ or nutrient‐specific association that remained significant after full adjustment and FDR correction. By contrast, Decay + Filling% showed weaker associations, likely because restored surfaces reflect past disease, treatment access and restorative history rather than current untreated disease alone. This finding is consistent with the established relevance of sugar exposure to untreated caries and supports the distinction between active decay and cumulative caries experience. Nevertheless, this finding should be interpreted cautiously, as two 24‐h recalls cannot fully capture habitual sugar frequency, food form, timing or long‐term exposure.

The explained‐variance analyses place these findings in a broader population context. Race and education level explained larger proportions of overall dietary variation than oral conditions, reflecting the strong social patterning of dietary intake. This is expected, as dietary behaviour is shaped by multiple upstream factors, including food access, cultural dietary patterns, income and education (Odoms‐Young et al. 2024; Olstad and McIntyre 2025). However, this does not diminish the relevance of oral‐health findings. Oral conditions were significantly associated with dietary profiles, and oral disease status explained the largest variation among oral factors, exceeding FTUs and tooth count. Our results also showed that oral conditions remained significantly associated with dietary intakes even after adjustment for socioeconomic factors. This distinction is important for practice: while socioeconomic determinants of diet require long‐term, multisectoral and policy‐level efforts, oral conditions are routinely identified and managed in dental settings. Although a causal direction cannot be inferred, oral‐health subgroups associated with less favourable dietary profiles may inform clinical attention to dietary patterns in adults with oral disease.

This study has several limitations. First, NHANES did not include direct assessment of occlusal contacts or masticatory performance. The FTU used here can only reflect potential posterior occlusal support rather than directly examined occlusal contacts or chewing function; it may not capture open bite, tooth mobility or other factors affecting occlusion and mastication. Second, the operational definition of caries warrants consideration. The untreated caries subgroup based on the presence of untreated coronal decay can only capture current disease but not cumulative caries experience, lesion activity, severity or treatment need. Accordingly, the untreated caries status should be interpreted as epidemiological oral disease subgroups rather than refined biological phenotypes. We included Decay + Filling% in the analyses of clinical extent to consider cumulative caries history. Third, exclusion of participants may have introduced selection bias. Included and excluded participants differed in several sociodemographic characteristics, suggesting that complete‐case selection was not entirely random. Therefore, the findings should be interpreted as primarily applying to adults with complete oral examination and reliable dietary recall data, and generalisability may be limited. Fourth, residual confounding is likely despite multivariable adjustment. Unmeasured factors, including dental care access, prosthesis use, xerostomia, food environment and health consciousness, may be related to both oral conditions and diet. Therefore, the coexisting untreated caries and periodontitis group may partly reflect a broader behavioural and social risk profile rather than oral‐disease‐specific associations alone. In addition, some food and nutrient variables were skewed, and regression‐based estimates may be influenced by extreme values, although outcomes were standardised to improve comparability across measures. Finally, the cross‐sectional design precludes establishing temporality or inferring causality, and the relationship between diet quality and oral health is likely bidirectional. In addition, although the measures used here are standardised and suitable for population‐based research, they cannot fully capture the complexity of oral function and context‐specific eating behaviours. Future longitudinal studies with more detailed clinical assessment are needed to clarify the interplay between oral conditions and dietary patterns.

## Author Contributions

Y.X. and M.B. contributed to study design, data analysis and interpretation, and led manuscript preparation. X.Y., J.S., H.L., Y.L., A.R. and C.Y. contributed to data analysis and interpretation. M.S.T. devised this study and contributed to protocol development, data interpretation and manuscript preparation. All authors critically revised the manuscript, gave their final approval and agreed to be accountable for all aspects of the work.

## Funding

This research was supported by the Clinical Research Program of 9th People's Hospital, Shanghai Jiao Tong University School of Medicine (grant number JYLJ202404, and JYLJ2025015), the National Natural Science Foundation of China (grant number 82301157) and the Cohort Development Program for Population, Disease‐Specific and Rare Disease Studies, Shanghai Ninth People's Hospital, Shanghai Jiao Tong University School of Medicine (grant number 2025DLB08).

## Conflicts of Interest

The authors declare no conflicts of interest.

## Supporting information


**Figure S1:** Flowchart of participant inclusion from NHANES 2011–2012 and 2013–2014.
**Figure S2:** Associations between tooth count and dietary intake patterns.
**Figure S3:** Associations between oral disease extent with food and nutrient intake in model 0 and model 1.
**Figure S4:** Variance in overall food and nutrient intake explained by oral conditions and participant characteristics in all included individuals.
**Table S1:** Demographic characteristics of included participants and excluded non‐pregnant adults with complete survey weight information in NHANES 2011–2014.
**Table S2:** Dietary indices, number of foods reported, food and nutrient intake across functional tooth unit categories.
**Table S3:** Characteristics of 6219 participants included in the present study classified by the numbers of tooth count.
**Table S4:** Dietary indices, number of foods reported, food and nutrient intake across oral conditions considering untreated caries and periodontitis in 5738 dentate individuals.

## Data Availability

Data sharing not applicable to this article as no datasets were generated or analysed during the current study.
