# Supplementary material for: Functional Tooth Units, Oral Disease Status and Dietary Quality in a Representative US Population
Source: J Clin Periodontol. 2026 Jul 13;53(10):1529–45. doi: 10.1111/jcpe.70165 (PMC13581468; doi:10.1111/jcpe.70165)
Supplement: Supplementary file 1 [file JCPE-53-1529-s001.docx]

**Supplementary Material**

**Functional Tooth Units, Oral Disease Status, and Dietary Quality in a Representative U.S. Population**

Yu Xie^1,2‡^, Mengning Bi^1,2‡^, Xiaoyu Yu^1,2^, Jie Shen^3,4^, Hairui Li^1,2^, Yuan Li^1,2^, Andrea Roccuzzo^1,2^, Changzheng Yuan^3,4^, Maurizio S. Tonetti^1,2,5*^

^1^ Shanghai PerioImplant Innovation Center, Institute for Oral, Craniofacial and Sensory Research, Ninth People's Hospital, Shanghai Jiao Tong University School of Medicine, Shanghai, China, 200125

^2^ National Clinical Research Center of Oral Diseases and National Center of Stomatology, College of Stomatology, Shanghai Jiao Tong University School of Medicine, Shanghai, China, 200125

^3^ School of Public Health, the Second Affiliated Hospital, Zhejiang University School of Medicine, Hangzhou, China

^4^Department of Nutrition, Harvard T.H. Chan School of Public Health, Boston, MA, USA

^5^ European Research Group on Periodontology (ERGOPerio), Genova, Italy

^‡^Equal contribution

*Corresponding author

**Correspondence**:

Maurizio S. Tonetti, Perio-Implant Innovation Center, 4F Building 1, 115 Jinzun Road, Pudong Research Campus, Shanghai Jiao Tong University School of Medicine, 200115, Shanghai, China.

email: maurizio.tonetti@ergoperio.eu

**Supplementary results:**

**Supplementary figures**

**Figure S1. Flowchart of participant inclusion from NHANES 2011-2012 and 2013-2014.**

Participants were initially identified from two NHANES cycles: 2011–2012 (n = 9,756) and 2013–2014 (n = 10,175), yielding a total of 19,931 participants. Sequential exclusion criteria were then applied. Participants younger than 18 years (n = 7,954), and those who reported being pregnant (n = 122) were excluded. For the objectives of this study, participants without a complete oral examination (n = 4,295), or without two reliable 24-hour dietary recalls (n = 1,246) were further excluded. In addition, 23 participants with root tips with restorative replacement were excluded because this condition is not clearly defined in the NHANES oral health examination data.

After these exclusions, 6,291 participants with complete tooth count, caries, periodontal examination data, and two reliable 24-hour dietary recalls were included in the final analysis.

**Figure S2.** **Associations between tooth count and dietary intake patterns.**

(A) Comparison of dietary indices and the number of fodds reported across tooth count status groups;

(B) Principal component analysis (PCA) of food and nutrient intake variables by tooth count status;

(C) Forest plot showing the associations of tooth count with dietary indices and the number of foods reported;

(D) Forest plot showing the associations of tooth count with selected food and nutrient intake variables.

All analyses accounted for the NHANES complex survey design. Tooth count was defined by considering natural teeth, dental implants, and fixed restorations. In (A), variables are presented as survey-weighted medians and interquartile ranges. Overall group differences were tested using survey-weighted Kruskal-Wallis rank tests, followed by survey-weighted Wilcoxon rank-sum tests with Bonferroni correction for post-hoc pairwise comparisons. Statistical significance is indicated as *: *p* < 0.05, **: *p* < 0.01, ***: *p* < 0.001.

For the PCA plots in (B), PCA was performed using food or nutrient intake variables that were energy-adjusted per 1,000 kcal and z-score standardized. Each point represents one participant, and ellipses indicate 95% confidence regions. Permutational multivariate analysis of variance (PERMANOVA) was used to estimate the explained variance (R²) of tooth count status in the PCA space. Explained variance refers to the proportion of statistical variation in PCA-derived dietary profiles associated with each factor in PERMANOVA and should not be interpreted causally. The food and nutrient variables included in the PCA are listed in Table S2.

For (C) and (D), associations were estimated using survey-weighted linear regression models. nif-FTUs was specified as exposures and variables of dietary intakes were specified as outcomes for statistical modelling and presentation. Model 0 was unadjusted; Model 1 was adjusted for age, sex, BMI, smoking status, diabetes status, and hypertension status; and Model 2 was additionally adjusted for race/ethnicity, education level, and income-poverty ratio. Beta estimates and 95% confidence intervals are shown as points and error bars. Associations were considered statistically significant when the 95% confidence interval did not cross the dashed line at beta = 0.

In (D), selected food and nutrient intake variables were defined as variables significantly associated with tooth count in the fully adjusted model (Model 2), with Benjamini-Hochberg false discovery rate (FDR) correction < 0.05. All food and nutrient intake variables were energy-adjusted using the per 1,000 kcal method and z-score standardized.

HEI2020, Healthy Eating Index 2020; DASH, Dietary Approaches to Stop Hypertension; MED, Mediterranean Diet Score; DII, Dietary Inflammatory Index.

**Figure S3. Associations between oral disease extent with food and nutrient intake in model 0 and model 1.**

(A) Clustered heatmaps showing the associations of oral disease extent, including Decay%, Decay + Filling%, CAL ≥ 1 mm%, CAL ≥ 3 mm%, and PD ≥ 4 mm%, with food intake variables in different models.

(B) Clustered heatmaps showing the associations of oral disease extent with nutrient intake variables in different adjustment models.

All analyses accounted for the NHANES complex survey design. Associations were estimated using survey-weighted linear regression models. Variables of oral disease extent were specified as exposures and variables of food and nutrient intakes as outcomes for statistical modelling and presentation. All food and nutrient intake variables were energy-adjusted using the per 1,000 kcal method and z-score standardized. Model 0 and 1 shown in this figure were the same as those used in Figure 2C. Model 0 was unadjusted survey-weighted linear regression model; and Model 1 was adjusted for nif-FTUs. Benjamini-Hochberg false discovery rate (FDR) correction were applied for multiple comparison, and statistical significance is indicated by *: FDR < 0.05.

FTU, functional tooth unit; nif, FTU definition considering natural teeth, dental implants, and fixed restorations; CAL, clinical attachment loss; PD, probing depth.

**Figure S4. Variance in overall food and nutrient intake explained by oral conditions and participant characteristics in all included individuals.**

The variance in overall food or nutrient intake explained by oral conditions, demographic factors, lifestyle factors, health status, and socio-economic factors was estimated using permutational multivariate analysis of variance (PERMANOVA) based on principal component analysis (PCA) of food and nutrient intake variables. Explained variance refers to the proportion of statistical variation in PCA-derived dietary profiles associated with each factor in PERMANOVA and should not be interpreted causally. PCA was performed using food or nutrient intake variables that were energy-adjusted per 1,000 kcal and z-score standardized. All oral conditions in all included individuals were defined according to tooth number, untreated caries and periodontitis status, resulting in five groups of edentulous, no untreated caries or periodontitis, untreated caries only, periodontitis only, and co-existing untreated caries and periodontitis. The food and nutrient variables included in the PCA are listed in Table S2.FTU-nif, functional tooth unit considering natural teeth, dental implants, and fixed restorations; Tooth-nif, tooth numbers considering natural teeth, dental implants, and fixed restorations.

**Supplementary tables**

**Table S1. Demographic characteristics of included participants and excluded non-pregnant adults with complete survey weight information in NHANES 2011–2014.**

| **Variables** | **All (n=9,204)** | **Excluded subjects** | **Included subjects** | ***p* value** |
| --- | --- | --- | --- | --- |
|  |  | **(n=2,913)** | **(n=6,291)** |  |
| **Age (yr)** | 47.0 (31.0 - 60.0) | 26.0 (21.0 - 46.0) | 51.0 (40.0 - 62.0) | **<0.001** |
| **Sex** |  |  |  | 0.606 |
| Male | 4421 (48.9%) | 1377 (49.5%) | 3044 (48.6%) |  |
| Female | 4783 (51.1%) | 1536 (50.5%) | 3247 (51.4%) |  |
| **Race** |  |  |  | **<0.001** |
| Mexican American | 1087 (9.0%) | 383 (11.7%) | 704 (7.8%) |  |
| Other Hispanic | 860 (5.7%) | 278 (6.4%) | 582 (5.4%) |  |
| Non-Hispanic White | 3788 (65.9%) | 1078 (60.5%) | 2710 (68.4%) |  |
| Non-Hispanic Black | 2157 (11.6%) | 721 (13.5%) | 1436 (10.7%) |  |
| Non-Hispanic Asian | 1025 (5.2%) | 336 (5.1%) | 689 (5.2%) |  |
| Other Race-Including Multi-Racial | 287 (2.6%) | 117 (2.9%) | 170 (2.5%) |  |
| **Education Level** |  |  |  | **<0.001** |
| Less than 9th grade | 644 (4.4%) | 132 (3.2%) | 512 (5.0%) |  |
| 9-11th grade | 1153 (9.8%) | 316 (9.8%) | 837 (9.9%) |  |
| High school graduate/GED or equivalent | 1884 (20.0%) | 498 (17.9%) | 1386 (20.9%) |  |
| Some college or AA degree | 2697 (31.2%) | 890 (33.5%) | 1807 (30.1%) |  |
| College graduate or above | 2306 (30.7%) | 560 (23.0%) | 1746 (34.2%) |  |
| Refused | 1 (0.0%) | 1 (0.1%) | 0 (0.0%) |  |
| Don't know | 5 (0.0%) | 2 (0.0%) | 3 (0.0%) |  |
| Missing | 514 (3.9%) | 514 (12.6%) | 0 (0.0%) |  |
| **Income-poverty ratio** | 2.6 (1.1 - 4.9) | 1.9 (0.8 - 4.0) | 2.9 (1.3 - 5.0) | **<0.001** |
| **BMI (kg/m^2^)** | 27.6 (23.9 - 32.3) | 26.4 (22.6 - 31.8) | 28.0 (24.6 - 32.5) | **<0.001** |

*Note:*

*BMI (body mass index): calculated as weight (kg) divided by height squared (m^2^);*

*Income-poverty ratio: calculated by dividing total family income by the poverty threshold based on poverty guidelines, specific to family size, year and state;*

*All estimates accounted for the NHANES complex sampling design, including sampling weights, strata, and primary sampling units.*

*Categorical variables are presented as unweighted counts and survey-weighted percentages. Continuous variables are presented as survey-weighted medians and interquartile ranges (IQRs). P values for categorical variables were calculated using survey-weighted chi-square tests, and P values for continuous variables were calculated using survey-weighted Wilcoxon rank-sum tests.*

*Excluded non-pregnant adults were defined as adult participants who were not pregnant but lacked key information required for the present analysis, including complete tooth count, caries examination, periodontal examination, or dietary recall data. Among 5,564 excluded non-pregnant adults, 2,651 did not have complete NHANES survey weight information. Therefore, only the remaining 2,913 excluded participants were included in this demographic comparison.*

**Table S2. Dietary indices, number of foods reported, food and nutrient intake across functional tooth unit categories.**

| **Dietary intake variable** | | **All (n=6,291)** | **Edentulous (n=553)** | **Impaired nif-FTUs  (n=2,174)** | **Adequate nif-FTUs  (n=3,564)** | **FDR** |
| --- | --- | --- | --- | --- | --- | --- |
| **Dietary indices** | |  |  |  |  |  |
|  | HEI2020 | 52.37 (44.14 - 60.76) | 48.66 (41.07 - 57.15) | 49.36 (42.34 - 57.87) | 53.75 (45.31 - 62.18) | **<0.001** |
|  | DASH | 22.50 (19.50 - 26.50) | 20.50 (17.50 - 23.50) | 21.50 (18.00 - 25.00) | 23.50 (20.00 - 27.50) | **<0.001** |
|  | MED | 3.50 (2.50 - 4.50) | 3.00 (2.50 - 4.00) | 3.00 (2.00 - 4.00) | 4.00 (3.00 - 5.00) | **<0.001** |
|  | DII | 1.93 (0.53 - 3.12) | 2.88 (1.67 - 3.97) | 2.42 (1.07 - 3.52) | 1.59 (0.22 - 2.90) | **<0.001** |
| **Number of foods reported** | | 16.50 (13.00 - 20.00) | 14.50 (11.50 - 18.50) | 15.50 (12.00 - 19.00) | 17.00 (14.00 - 20.50) | **<0.001** |
| **Food patterns** | |  |  |  |  |  |
|  | Citrus, melons, and berries (cup eq./1000 kcal) | 0.00 (0.00 - 0.13) | 0.00 (0.00 - 0.04) | 0.00 (0.00 - 0.08) | 0.01 (0.00 - 0.15) | **<0.001** |
|  | Other fruits (cup eq./1000 kcal) | 0.13 (0.00 - 0.41) | 0.02 (0.00 - 0.33) | 0.09 (0.00 - 0.36) | 0.16 (0.00 - 0.44) | **<0.001** |
|  | Fruit juice (cup eq./1000 kcal) | 0.00 (0.00 - 0.15) | 0.00 (0.00 - 0.21) | 0.00 (0.00 - 0.15) | 0.00 (0.00 - 0.14) | 0.923 |
|  | Total fruit (cup eq./1000 kcal) | 0.36 (0.07 - 0.75) | 0.24 (0.01 - 0.61) | 0.31 (0.03 - 0.71) | 0.39 (0.11 - 0.78) | **<0.001** |
|  | Dark green vegetables (cup eq./1000 kcal) | 0.00 (0.00 - 0.11) | 0.00 (0.00 - 0.00) | 0.00 (0.00 - 0.03) | 0.00 (0.00 - 0.15) | **<0.001** |
|  | Tomatoes (cup eq./1000 kcal) | 0.11 (0.03 - 0.21) | 0.06 (0.00 - 0.15) | 0.10 (0.03 - 0.20) | 0.11 (0.04 - 0.22) | **<0.001** |
|  | Other red and orange vegetables (cup eq./1000 kcal) | 0.01 (0.00 - 0.07) | 0.00 (0.00 - 0.04) | 0.00 (0.00 - 0.05) | 0.01 (0.00 - 0.08) | **<0.001** |
|  | Total red and orange vegetables (cup eq./1000 kcal) | 0.15 (0.07 - 0.29) | 0.10 (0.02 - 0.22) | 0.14 (0.05 - 0.27) | 0.17 (0.08 - 0.30) | **<0.001** |
|  | Potatoes (white potatoes) (cup eq./1000 kcal) | 0.11 (0.00 - 0.26) | 0.15 (0.00 - 0.35) | 0.12 (0.00 - 0.29) | 0.09 (0.00 - 0.24) | **0.006** |
|  | Other starchy vegetables (cup eq./1000 kcal) | 0.00 (0.00 - 0.04) | 0.00 (0.00 - 0.06) | 0.00 (0.00 - 0.04) | 0.00 (0.00 - 0.04) | 0.563 |
|  | Total starchy vegetables (cup eq./1000 kcal) | 0.15 (0.00 - 0.31) | 0.20 (0.03 - 0.42) | 0.16 (0.00 - 0.33) | 0.14 (0.00 - 0.30) | **<0.001** |
|  | Other vegetables (cup eq./1000 kcal) | 0.23 (0.09 - 0.43) | 0.15 (0.04 - 0.36) | 0.18 (0.06 - 0.39) | 0.25 (0.11 - 0.45) | **<0.001** |
|  | Total vegetables (cup eq./1000 kcal) | 0.72 (0.45 - 1.08) | 0.65 (0.36 - 0.99) | 0.64 (0.39 - 1.03) | 0.76 (0.49 - 1.13) | **<0.001** |
|  | Beans and peas computed as vegetables (cup eq./1000 kcal) | 0.00 (0.00 - 0.07) | 0.00 (0.00 - 0.04) | 0.00 (0.00 - 0.07) | 0.00 (0.00 - 0.08) | 0.121 |
|  | Whole grains (oz. eq./1000 kcal) | 0.31 (0.00 - 0.79) | 0.23 (0.00 - 0.79) | 0.23 (0.00 - 0.67) | 0.34 (0.03 - 0.83) | **0.003** |
|  | Refined grains (oz. eq./1000 kcal) | 2.51 (1.81 - 3.35) | 2.28 (1.67 - 3.26) | 2.53 (1.83 - 3.38) | 2.51 (1.81 - 3.35) | 0.392 |
|  | Total grains (oz. eq./1000 kcal) | 3.08 (2.33 - 3.86) | 2.98 (2.22 - 3.68) | 3.04 (2.29 - 3.86) | 3.10 (2.36 - 3.86) | 0.343 |
|  | Meat (oz. eq./1000 kcal) | 0.56 (0.00 - 1.20) | 0.73 (0.01 - 1.43) | 0.59 (0.00 - 1.31) | 0.53 (0.00 - 1.15) | **0.040** |
|  | Cured meat (oz. eq./1000 kcal) | 0.25 (0.00 - 0.71) | 0.31 (0.00 - 0.78) | 0.28 (0.00 - 0.77) | 0.24 (0.00 - 0.68) | 0.427 |
|  | Organ meat (oz. eq./1000 kcal) | 0.00 (0.00 - 0.00) | 0.00 (0.00 - 0.00) | 0.00 (0.00 - 0.00) | 0.00 (0.00 - 0.00) | 0.283 |
|  | Poultry (oz. eq./1000 kcal) | 0.49 (0.00 - 1.14) | 0.32 (0.00 - 1.20) | 0.42 (0.00 - 1.10) | 0.51 (0.00 - 1.15) | 0.061 |
|  | Seafood high in n-3 fatty acids (oz. eq./1000 kcal) | 0.00 (0.00 - 0.00) | 0.00 (0.00 - 0.00) | 0.00 (0.00 - 0.00) | 0.00 (0.00 - 0.00) | 0.359 |
|  | Seafood low in n-3 fatty acids (oz. eq./1000 kcal) | 0.00 (0.00 - 0.11) | 0.00 (0.00 - 0.00) | 0.00 (0.00 - 0.00) | 0.00 (0.00 - 0.15) | 0.359 |
|  | Total meat, poultry, and seafood (oz. eq./1000 kcal) | 2.16 (1.44 - 3.11) | 2.27 (1.44 - 3.34) | 2.18 (1.45 - 3.13) | 2.13 (1.43 - 3.07) | 0.121 |
|  | Eggs (oz. eq./1000 kcal) | 0.12 (0.02 - 0.42) | 0.17 (0.03 - 0.46) | 0.14 (0.02 - 0.48) | 0.11 (0.02 - 0.40) | 0.096 |
|  | Soybean products (oz. eq./1000 kcal) | 0.00 (0.00 - 0.00) | 0.00 (0.00 - 0.00) | 0.00 (0.00 - 0.00) | 0.00 (0.00 - 0.00) | **0.016** |
|  | Nuts and seeds (oz. eq./1000 kcal) | 0.08 (0.00 - 0.47) | 0.00 (0.00 - 0.19) | 0.00 (0.00 - 0.22) | 0.13 (0.00 - 0.60) | **<0.001** |
|  | Beans and peas computed as protein foods (oz. eq./1000 kcal) | 0.00 (0.00 - 0.30) | 0.00 (0.00 - 0.17) | 0.00 (0.00 - 0.28) | 0.00 (0.00 - 0.31) | 0.121 |
|  | Total protein foods (oz. eq./1000 kcal) | 2.82 (2.02 - 3.84) | 2.73 (2.02 - 3.90) | 2.75 (1.97 - 3.71) | 2.86 (2.06 - 3.88) | 0.229 |
|  | Milk (cup eq./1000 kcal) | 0.22 (0.06 - 0.51) | 0.27 (0.08 - 0.57) | 0.22 (0.06 - 0.50) | 0.21 (0.06 - 0.50) | **0.034** |
|  | Yogurt (cup eq./1000 kcal) | 0.00 (0.00 - 0.00) | 0.00 (0.00 - 0.00) | 0.00 (0.00 - 0.00) | 0.00 (0.00 - 0.00) | **<0.001** |
|  | Cheese (cup eq./1000 kcal) | 0.27 (0.07 - 0.53) | 0.19 (0.00 - 0.44) | 0.24 (0.03 - 0.47) | 0.30 (0.09 - 0.55) | **<0.001** |
|  | Total dairy (cup eq./1000 kcal) | 0.68 (0.39 - 1.02) | 0.61 (0.38 - 0.98) | 0.64 (0.35 - 0.98) | 0.70 (0.41 - 1.03) | 0.129 |
|  | Oils (g/1000 kcal) | 11.50 (7.85 - 15.81) | 10.37 (7.16 - 14.51) | 10.69 (7.03 - 14.82) | 11.98 (8.34 - 16.34) | **<0.001** |
|  | Solid fats (g/1000 kcal) | 15.88 (11.39 - 20.52) | 16.71 (11.81 - 21.01) | 16.31 (11.81 - 20.99) | 15.63 (11.19 - 20.27) | **0.014** |
|  | Added sugars (tsp. eq./1000 kcal) | 6.63 (4.11 - 10.01) | 8.41 (4.73 - 12.98) | 7.24 (4.27 - 11.31) | 6.33 (3.94 - 9.42) | **<0.001** |
|  | Alcoholic drinks (no. of drinks/1000 kcal) | 0.00 (0.00 - 0.34) | 0.00 (0.00 - 0.00) | 0.00 (0.00 - 0.20) | 0.00 (0.00 - 0.40) | **<0.001** |
| **Nutrients** | |  |  |  |  |  |
|  | Protein intake (gm/1000 kcal) | 38.94 (33.01 - 45.95) | 39.05 (30.99 - 46.92) | 37.81 (31.91 - 45.10) | 39.42 (33.59 - 46.30) | **0.014** |
|  | Carbohydrate intake (gm/1000 kcal) | 120.89 (105.90 - 135.72) | 126.22 (107.47 - 140.31) | 122.58 (107.73 - 139.11) | 119.85 (104.68 - 133.90) | **<0.001** |
|  | Total sugars intake (gm/1000 kcal) | 50.02 (36.72 - 64.53) | 56.40 (40.71 - 74.09) | 52.48 (37.23 - 68.47) | 48.87 (36.14 - 61.93) | **<0.001** |
|  | Dietary fiber intake (gm/1000 kcal) | 8.30 (6.20 - 10.91) | 7.17 (4.97 - 9.49) | 7.64 (5.66 - 10.00) | 8.69 (6.63 - 11.28) | **<0.001** |
|  | Total fat intake (gm/1000 kcal) | 37.99 (32.56 - 42.87) | 37.68 (31.58 - 42.78) | 37.19 (32.00 - 42.64) | 38.18 (32.99 - 42.98) | 0.081 |
|  | Total saturated fatty acids (gm/1000 kcal) | 11.92 (9.76 - 14.28) | 11.84 (9.65 - 14.33) | 11.93 (9.67 - 14.33) | 11.92 (9.82 - 14.23) | 0.979 |
|  | Total monounsaturated fatty acids (gm/1000 kcal) | 13.17 (11.01 - 15.32) | 13.20 (10.97 - 15.09) | 12.92 (10.87 - 15.19) | 13.29 (11.05 - 15.41) | 0.216 |
|  | Total polyunsaturated fatty acids (gm/1000 kcal) | 8.69 (6.93 - 10.58) | 8.51 (6.51 - 10.25) | 8.40 (6.56 - 10.26) | 8.87 (7.18 - 10.73) | **<0.001** |
|  | Cholesterol (mg/1000 kcal) | 124.64 (87.59 - 173.27) | 134.57 (93.01 - 192.40) | 131.79 (88.26 - 180.33) | 121.16 (86.99 - 169.04) | **0.008** |
|  | Alcohol (gm/1000 kcal) | 0.00 (0.00 - 4.73) | 0.00 (0.00 - 0.00) | 0.00 (0.00 - 2.82) | 0.00 (0.00 - 5.56) | **<0.001** |
|  | Retinol (mcg/1000 kcal) | 181.22 (113.78 - 262.56) | 183.95 (122.62 - 297.82) | 180.19 (110.93 - 263.20) | 181.34 (113.83 - 261.42) | 0.596 |
|  | Vitamin A, RAE (mcg/1000 kcal) | 273.37 (189.53 - 418.94) | 246.30 (174.96 - 395.07) | 255.55 (172.95 - 384.01) | 285.85 (199.15 - 426.88) | **<0.001** |
|  | Vitamin D (D2+D3) (mcg/1000 kcal) | 1.79 (0.96 - 2.94) | 1.98 (1.25 - 3.34) | 1.79 (1.00 - 3.06) | 1.76 (0.93 - 2.86) | **0.011** |
|  | Vitamin E as alpha-tocopherol (mg/1000 kcal) | 3.84 (2.98 - 5.15) | 3.53 (2.90 - 4.52) | 3.59 (2.70 - 4.64) | 4.04 (3.13 - 5.47) | **<0.001** |
|  | Added alpha-tocopherol (Vitamin E) (mg/1000 kcal) | 0.00 (0.00 - 0.00) | 0.00 (0.00 - 0.00) | 0.00 (0.00 - 0.00) | 0.00 (0.00 - 0.00) | 0.027 |
|  | Vitamin K (mcg/1000 kcal) | 41.05 (26.61 - 73.50) | 37.70 (23.66 - 61.07) | 35.96 (24.07 - 56.93) | 45.33 (27.87 - 82.42) | **<0.001** |
|  | Alpha-carotene (mcg/1000 kcal) | 50.81 (14.27 - 259.49) | 48.28 (10.56 - 199.07) | 36.44 (10.28 - 189.40) | 60.44 (16.85 - 318.18) | **0.002** |
|  | Beta-carotene (mcg/1000 kcal) | 636.58 (255.47 - 1581.58) | 390.57 (198.37 - 982.37) | 455.15 (183.65 - 1157.02) | 771.73 (304.62 - 1839.66) | **<0.001** |
|  | Beta-cryptoxanthin (mcg/1000 kcal) | 21.16 (7.87 - 51.40) | 18.47 (6.13 - 46.69) | 20.22 (7.21 - 52.10) | 21.90 (8.29 - 52.20) | 0.212 |
|  | Thiamin (Vitamin B1) (mg/1000 kcal) | 0.76 (0.62 - 0.92) | 0.75 (0.61 - 0.93) | 0.75 (0.62 - 0.93) | 0.76 (0.63 - 0.92) | 0.755 |
|  | Riboflavin (Vitamin B2) (mg/1000 kcal) | 1.00 (0.79 - 1.23) | 1.00 (0.83 - 1.31) | 0.98 (0.76 - 1.21) | 1.00 (0.81 - 1.23) | 0.144 |
|  | Niacin (mg/1000 kcal) | 11.81 (9.78 - 14.53) | 11.89 (9.57 - 14.82) | 11.57 (9.64 - 14.17) | 11.87 (9.88 - 14.64) | 0.144 |
|  | Vitamin B6 (mg/1000 kcal) | 0.96 (0.76 - 1.25) | 0.91 (0.70 - 1.28) | 0.93 (0.73 - 1.20) | 0.98 (0.77 - 1.27) | **0.003** |
|  | Total Folate (mcg/1000 kcal) | 186.13 (144.83 - 243.05) | 173.11 (134.81 - 235.92) | 176.54 (138.20 - 229.24) | 192.01 (150.95 - 247.29) | **<0.001** |
|  | Folic acid (mcg/1000 kcal) | 72.50 (46.22 - 113.69) | 69.81 (40.12 - 110.14) | 72.40 (47.44 - 110.79) | 72.64 (45.96 - 114.32) | 0.647 |
|  | Food folate (mcg/1000 kcal) | 102.65 (78.46 - 135.28) | 96.63 (72.64 - 123.22) | 95.08 (71.08 - 125.03) | 107.69 (82.14 - 139.79) | **<0.001** |
|  | Folate DFE (mcg/1000 kcal) | 240.60 (182.78 - 317.05) | 223.37 (164.01 - 306.70) | 225.96 (177.34 - 304.74) | 245.96 (187.32 - 323.75) | **0.013** |
|  | Vitamin B12 (mcg/1000 kcal) | 2.06 (1.40 - 2.92) | 2.31 (1.54 - 3.33) | 2.02 (1.36 - 2.98) | 2.07 (1.41 - 2.88) | **0.024** |
|  | Added vitamin B12 (mcg/1000 kcal) | 0.00 (0.00 - 0.68) | 0.00 (0.00 - 0.75) | 0.00 (0.00 - 0.56) | 0.00 (0.00 - 0.69) | 0.108 |
|  | Vitamin C (mg/1000 kcal) | 32.19 (15.37 - 58.54) | 25.22 (11.72 - 50.29) | 29.64 (13.05 - 58.35) | 33.65 (16.94 - 59.32) | **0.002** |
|  | Total choline (mg/1000 kcal) | 154.37 (125.34 - 192.15) | 161.96 (126.84 - 207.84) | 157.57 (127.59 - 192.56) | 152.37 (124.60 - 190.78) | 0.121 |
|  | Calcium (mg/1000 kcal) | 445.67 (340.43 - 568.72) | 432.32 (327.35 - 540.16) | 432.01 (325.34 - 557.16) | 452.74 (351.45 - 575.04) | 0.081 |
|  | Phosphorus (mg/1000 kcal) | 661.94 (573.55 - 763.69) | 662.60 (546.51 - 762.80) | 641.65 (555.01 - 749.37) | 668.49 (582.34 - 771.01) | **0.002** |
|  | Magnesium (mg/1000 kcal) | 146.26 (118.90 - 178.38) | 130.41 (106.73 - 157.80) | 136.06 (111.76 - 167.41) | 150.68 (124.09 - 184.06) | **<0.001** |
|  | Sodium (mg/1000 kcal) | 1653.29 (1418.82 - 1927.51) | 1699.45 (1408.14 - 1989.15) | 1640.29 (1402.75 - 1934.68) | 1653.74 (1427.76 - 1914.07) | 0.603 |
|  | Potassium (mg/1000 kcal) | 1319.93 (1095.85 - 1577.03) | 1289.25 (1040.02 - 1547.24) | 1273.18 (1053.75 - 1540.78) | 1338.71 (1114.81 - 1595.59) | **0.001** |
|  | Iron (mg/1000 kcal) | 6.76 (5.59 - 8.50) | 6.63 (5.52 - 8.66) | 6.68 (5.36 - 8.45) | 6.81 (5.64 - 8.55) | 0.393 |
|  | Zinc (mg/1000 kcal) | 5.14 (4.23 - 6.33) | 5.11 (3.95 - 6.39) | 5.00 (4.10 - 6.19) | 5.21 (4.32 - 6.38) | **0.027** |
|  | Copper (mg/1000 kcal) | 0.59 (0.48 - 0.74) | 0.53 (0.44 - 0.66) | 0.55 (0.43 - 0.69) | 0.61 (0.50 - 0.76) | **<0.001** |
|  | Selenium (mcg/1000 kcal) | 54.09 (45.42 - 64.65) | 53.48 (42.21 - 66.56) | 53.90 (45.01 - 64.61) | 54.25 (45.80 - 64.54) | 0.647 |
|  | Lycopene (mcg/1000 kcal) | 1344.36 (407.54 - 3225.89) | 735.15 (124.33 - 2298.82) | 1238.38 (337.83 - 3004.60) | 1458.58 (473.24 - 3381.22) | **<0.001** |
|  | Lutein + zeaxanthin (mcg/1000 kcal) | 461.54 (254.82 - 927.05) | 403.09 (216.09 - 730.16) | 401.60 (215.87 - 689.45) | 495.75 (285.01 - 1098.11) | **<0.001** |
|  | Caffeine (mg/1000 kcal) | 66.16 (23.81 - 124.97) | 72.69 (34.12 - 151.31) | 66.16 (25.56 - 129.22) | 64.60 (22.46 - 121.26) | **0.032** |
|  | Theobromine (mg/1000 kcal) | 7.94 (0.00 - 24.21) | 5.83 (0.00 - 21.17) | 6.14 (0.00 - 23.76) | 8.96 (0.00 - 24.78) | **0.004** |
|  | Total plain water drank yesterday (gm/1000 kcal) | 436.56 (179.82 - 817.84) | 252.06 (52.29 - 613.46) | 362.21 (129.66 - 746.56) | 484.06 (226.25 - 858.55) | **<0.001** |
|  | Total tap water drank yesterday (gm/1000 kcal) | 184.63 (0.00 - 528.32) | 38.65 (0.00 - 298.10) | 109.77 (0.00 - 398.28) | 240.70 (0.00 - 604.38) | **<0.001** |
|  | Total bottled water drank (gm/1000 kcal) | 0.00 (0.00 - 275.99) | 0.00 (0.00 - 200.56) | 0.00 (0.00 - 297.98) | 0.00 (0.00 - 274.91) | 0.325 |
|  | Moisture (gm/1000 kcal) | 1373.69 (1058.08 - 1823.78) | 1313.08 (992.65 - 1711.11) | 1335.96 (1009.94 - 1813.76) | 1388.42 (1091.22 - 1842.72) | **0.002** |
|  | SFA 4:0 (Butanoic) (gm/1000 kcal) | 0.21 (0.13 - 0.33) | 0.19 (0.10 - 0.31) | 0.21 (0.11 - 0.31) | 0.22 (0.13 - 0.34) | **0.045** |
|  | SFA 6:0 (Hexanoic) (gm/1000 kcal) | 0.13 (0.07 - 0.19) | 0.12 (0.07 - 0.18) | 0.13 (0.07 - 0.19) | 0.13 (0.08 - 0.20) | 0.431 |
|  | SFA 8:0 (Octanoic) (gm/1000 kcal) | 0.10 (0.07 - 0.15) | 0.09 (0.06 - 0.16) | 0.10 (0.06 - 0.15) | 0.10 (0.07 - 0.15) | 0.325 |
|  | SFA 10:0 (Decanoic) (gm/1000 kcal) | 0.21 (0.13 - 0.30) | 0.19 (0.11 - 0.28) | 0.20 (0.12 - 0.30) | 0.21 (0.14 - 0.31) | 0.056 |
|  | SFA 12:0 (Dodecanoic) (gm/1000 kcal) | 0.29 (0.17 - 0.45) | 0.25 (0.15 - 0.42) | 0.28 (0.16 - 0.44) | 0.29 (0.18 - 0.46) | 0.059 |
|  | SFA 14:0 (Tetradecanoic) (gm/1000 kcal) | 0.95 (0.64 - 1.31) | 0.88 (0.58 - 1.29) | 0.91 (0.64 - 1.30) | 0.97 (0.65 - 1.31) | 0.212 |
|  | SFA 16:0 (Hexadecanoic) (gm/1000 kcal) | 6.45 (5.42 - 7.65) | 6.63 (5.43 - 7.66) | 6.47 (5.39 - 7.71) | 6.42 (5.42 - 7.61) | 0.704 |
|  | SFA 18:0 (Octadecanoic) (gm/1000 kcal) | 2.92 (2.37 - 3.53) | 3.03 (2.40 - 3.60) | 2.95 (2.40 - 3.57) | 2.90 (2.35 - 3.51) | 0.339 |
|  | MFA 16:1 (Hexadecenoic) (gm/1000 kcal) | 0.46 (0.35 - 0.61) | 0.47 (0.36 - 0.62) | 0.48 (0.36 - 0.62) | 0.46 (0.35 - 0.60) | 0.248 |
|  | MFA 18:1 (Octadecenoic) (gm/1000 kcal) | 12.08 (10.02 - 14.17) | 12.06 (10.02 - 13.73) | 11.89 (9.89 - 13.93) | 12.19 (10.10 - 14.28) | 0.061 |
|  | MFA 20:1 (Eicosenoic) (gm/1000 kcal) | 0.13 (0.09 - 0.17) | 0.12 (0.10 - 0.17) | 0.13 (0.09 - 0.17) | 0.13 (0.09 - 0.17) | 0.979 |
|  | MFA 22:1 (Docosenoic) (gm/1000 kcal) | 0.01 (0.00 - 0.01) | 0.01 (0.00 - 0.01) | 0.01 (0.00 - 0.01) | 0.01 (0.00 - 0.02) | 0.515 |
|  | PFA 18:2 (Octadecadienoic) (gm/1000 kcal) | 7.70 (6.04 - 9.41) | 7.52 (5.75 - 9.05) | 7.38 (5.69 - 9.07) | 7.81 (6.25 - 9.49) | **<0.001** |
|  | PFA 18:3 (Octadecatrienoic) (gm/1000 kcal) | 0.78 (0.60 - 1.01) | 0.77 (0.58 - 1.00) | 0.75 (0.56 - 0.99) | 0.79 (0.61 - 1.01) | **0.006** |
|  | PFA 18:4 (Octadecatetraenoic) (gm/1000 kcal) | 0.00 (0.00 - 0.00) | 0.00 (0.00 - 0.00) | 0.00 (0.00 - 0.00) | 0.00 (0.00 - 0.00) | 0.407 |
|  | PFA 20:4 (Eicosatetraenoic) (gm/1000 kcal) | 0.06 (0.04 - 0.10) | 0.07 (0.05 - 0.11) | 0.07 (0.04 - 0.10) | 0.06 (0.04 - 0.09) | **0.001** |
|  | PFA 20:5 (Eicosapentaenoic) (gm/1000 kcal) | 0.00 (0.00 - 0.01) | 0.00 (0.00 - 0.01) | 0.00 (0.00 - 0.01) | 0.00 (0.00 - 0.01) | 0.438 |
|  | PFA 22:5 (Docosapentaenoic) (gm/1000 kcal) | 0.01 (0.01 - 0.01) | 0.01 (0.01 - 0.01) | 0.01 (0.01 - 0.01) | 0.01 (0.01 - 0.01) | 0.808 |
|  | PFA 22:6 (Docosahexaenoic) (gm/1000 kcal) | 0.01 (0.00 - 0.03) | 0.01 (0.00 - 0.03) | 0.01 (0.00 - 0.03) | 0.01 (0.00 - 0.03) | 0.864 |

*Note:*

*nif-FTU refers to functional tooth unit considering natural teeth, dental implants, and fixed restorations; HEI2020, Healthy Eating Index 2020; DASH, Dietary Approaches to Stop Hypertension Index; MED, alternate Mediterranean Diet Score; DII, Dietary Inflammatory Index; IQR, interquartile range; FDR, false discovery rate.*

*All included participants had two reliable 24-hour dietary recalls. Dietary indices, the number of foods reported, and food and nutrient intake variables were calculated as the average of the two recall days. Food and nutrient intake variables were energy-adjusted using the per 1,000 kcal method.* *The food and nutrient intake variables listed in this table were also used in the PCA analyses.*

*All estimates accounted for the NHANES complex sampling design, including sampling weights, strata, and primary sampling units. Variables are presented as survey-weighted medians and interquartile ranges (IQRs). P values were calculated using survey-weighted Kruskal-Wallis rank tests. Benjamini-Hochberg FDR correction was applied for multiple comparisons.*

**Table S3. Characteristics of 6,219 participants included in the present study classified by the numbers of tooth count.**

| **Variables** | **All (n=6,291)** | **Edentulous** | **Impaired tooth numbers  (nif-Tooth counts = 1-19)** | **Adequate tooth numbers**  **(nif-Tooth count ≥20)** | ***p* value** |
| --- | --- | --- | --- | --- | --- |
|  |  | **(n=553)** | **(n=1,052)** | **(n=4,686)** |  |
| **Age (yr)** | 51.0 (40.0 - 62.0) | 68.0 (57.0 - 76.0) | 60.0 (52.0 - 69.0) | 49.0 (39.0 - 59.0) | **<0.001** |
| **Sex** |  |  |  |  | 0.769 |
| Male | 3044 (48.6%) | 282 (50.4%) | 517 (49.1%) | 2245 (48.3%) |  |
| Female | 3247 (51.4%) | 271 (49.6%) | 535 (50.9%) | 2441 (51.7%) |  |
| **Race** |  |  |  |  | **<0.001** |
| Mexican American | 704 (7.8%) | 23 (2.5%) | 101 (6.7%) | 580 (8.4%) |  |
| Other Hispanic | 582 (5.4%) | 43 (4.3%) | 105 (5.9%) | 434 (5.4%) |  |
| Non-Hispanic White | 2710 (68.4%) | 274 (71.7%) | 392 (62.3%) | 2044 (69.1%) |  |
| Non-Hispanic Black | 1436 (10.7%) | 163 (13.9%) | 375 (19.2%) | 898 (9.1%) |  |
| Non-Hispanic Asian | 689 (5.2%) | 26 (2.1%) | 56 (3.5%) | 607 (5.7%) |  |
| Other Race-Including Multi-Racial | 170 (2.5%) | 24 (5.4%) | 23 (2.3%) | 123 (2.3%) |  |
| **Education Level** | 0 (0.0%) | 0 (0.0%) | 0 (0.0%) | 0 (0.0%) | **<0.001** |
| Less than 9th grade | 512 (5.0%) | 92 (11.7%) | 142 (10.0%) | 278 (3.6%) |  |
| 9-11th grade | 837 (9.9%) | 153 (25.4%) | 217 (17.8%) | 467 (7.4%) |  |
| High school graduate/GED or equivalent | 1386 (20.9%) | 155 (30.6%) | 325 (35.0%) | 906 (17.9%) |  |
| Some college or AA degree | 1807 (30.1%) | 117 (24.8%) | 268 (27.2%) | 1422 (31.0%) |  |
| College graduate or above | 1746 (34.2%) | 36 (7.4%) | 99 (10.0%) | 1611 (40.1%) |  |
| Don't know | 3 (0.0%) | 0 (0.0%) | 1 (0.0%) | 2 (0.0%) |  |
| **Income-poverty ratio** | 2.9 (1.3 - 5.0) | 1.6 (0.9 - 2.7) | 1.6 (0.9 - 2.9) | 3.4 (1.5 - 5.0) | **<0.001** |
| **BMI (kg/m^2^)** | 28.0 (24.6 - 32.5) | 28.4 (24.5 - 33.0) | 29.0 (25.1 - 33.8) | 27.8 (24.6 - 32.2) | 0.055 |
| **Energy intake (kcal)** | 1973.5 (1563.0 - 2492.0) | 1729.0 (1318.0 - 2288.5) | 1873.0 (1444.0 - 2388.0) | 2008.5 (1597.5 - 2518.5) | **<0.001** |
| **nif-Tooth count** | 26.0 (22.0 - 28.0) | 0.0 (0.0 - 0.0) | 13.0 (8.0 - 17.0) | 27.0 (25.0 - 28.0) | **<0.001** |
| **nif-FTU** | 10.0 (4.0 - 12.0) | 0.0 (0.0 - 0.0) | 0.0 (0.0 - 2.0) | 11.0 (8.0 - 12.0) | **<0.001** |
| **Alcohol consumption** |  |  |  |  | **<0.001** |
| Everyday | 260 (5.2%) | 18 (2.3%) | 56 (5.6%) | 186 (5.4%) |  |
| Every week | 1619 (30.8%) | 75 (14.4%) | 174 (18.0%) | 1370 (34.2%) |  |
| Every month/year | 959 (17.0%) | 35 (5.6%) | 101 (10.5%) | 823 (18.9%) |  |
| Several month/year | 1178 (17.0%) | 111 (22.3%) | 210 (19.3%) | 857 (16.2%) |  |
| Never | 1152 (15.2%) | 212 (35.4%) | 305 (29.3%) | 635 (11.3%) |  |
| Missing | 1123 (14.9%) | 102 (19.9%) | 206 (17.2%) | 815 (14.1%) |  |
| **Smoking status** |  |  |  |  | **<0.001** |
| Never | 3450 (55.0%) | 169 (30.1%) | 423 (35.5%) | 2858 (60.1%) |  |
| Former smoker | 1667 (26.9%) | 212 (38.1%) | 321 (30.6%) | 1134 (25.4%) |  |
| Current smoker | 1170 (18.1%) | 172 (31.8%) | 307 (33.9%) | 691 (14.5%) |  |
| Missing | 4 (0.0%) | 0 (0.0%) | 1 (0.0%) | 3 (0.0%) |  |
| **Blood pressure status** |  |  |  |  | **<0.001** |
| Normal | 2539 (42.7%) | 159 (31.8%) | 269 (28.6%) | 2111 (45.8%) |  |
| Elevated | 1064 (17.5%) | 89 (16.9%) | 199 (19.2%) | 776 (17.3%) |  |
| Hypertension stage 1 | 1349 (21.4%) | 118 (21.4%) | 260 (22.0%) | 971 (21.3%) |  |
| Hypertension stage 2 | 1168 (16.1%) | 162 (25.8%) | 273 (25.2%) | 733 (13.9%) |  |
| Hypertension Crisis | 45 (0.4%) | 11 (0.9%) | 21 (2.0%) | 13 (0.2%) |  |
| Missing | 126 (1.8%) | 14 (3.2%) | 30 (3.0%) | 82 (1.5%) |  |
| **Diabetes status** |  |  |  |  | **<0.001** |
| Non-diabetes | 3384 (61.8%) | 199 (44.4%) | 388 (39.2%) | 2797 (66.8%) |  |
| Prediabetes | 1940 (27.2%) | 218 (36.3%) | 418 (42.4%) | 1304 (24.1%) |  |
| Diabetes | 512 (5.5%) | 72 (11.6%) | 140 (9.3%) | 300 (4.4%) |  |
| Uncontrolled diabetes | 280 (3.2%) | 44 (5.8%) | 69 (5.8%) | 167 (2.6%) |  |
| Missing | 175 (2.3%) | 20 (1.9%) | 37 (3.4%) | 118 (2.1%) |  |

*Note:*

*nif-Tooth count refers to thenumber of teeth counted by considering natural teeth, dental implants, and fixed restorations; nif-FTU refers to functional tooth unit considering natural teeth, dental implants, and fixed restorations; BMI (body mass index): calculated as weight (kg) divided by height squared (m^2^);*

*Income-poverty ratio: calculated by dividing total family income by the poverty threshold based on poverty guidelines, specific to family size, year and state;*

*Smoking status: non-smoker, smoked <100 cigarettes in life; former smoker, smoked ≥100 cigarettes in life & quitted from smoking; and current smoker, smoked ≥100 cigarettes in life & currently smoking;*

*Blood pressure status: categorized based on examined systolic and diastolic blood pressure values (SBP and DBP); Normal, SBP <120 mmHg and DBP <80 mmHg; Elevated, SBP 120-129 mmHg and DBP <80 mmHg; hypertension stage I, SBP 130-139 mmHg or DBP 80-89 mmHg; hypertension stage II, SBP ≥140 mmHg or DBP ≥90 mmHg; hypertension crisis, SBP >180 mmHg and/or DBP >120 mmHg;*

*Diabetes status: defined by tested glycohemoglobin (HbA1c) levels. Non-diabetes, HbA1c <5.7%; Prediabetes, HbA1c 5.7-6.4%; Diabetes, HbA1c ≥6.5%; Poorly controlled diabetes, HbA1c ≥8.0%.*

*All estimates accounted for the NHANES complex sampling design, including sampling weights, strata, and primary sampling units.*

*Categorical variables are presented as unweighted counts and survey-weighted percentages. Continuous variables are presented as survey-weighted medians and interquartile ranges (IQRs). P values for categorical variables were calculated using survey-weighted chi-square tests, and P values for continuous variables were calculated using survey-weighted Kruskal-Wallis rank tests.*

**Table S4. Dietary indices, number of foods reported, food and nutrient intake across oral conditions considering untreated caries and periodontitis in 5,738 dentate individuals.**

| **Dietary intake variable** | | **All (n=5,738)** | **No untreated caries/periodontitis (n=2,455)** | **untreated Caries only (n=561)** | **Periodontitis only (n=1,731)** | **untreated Caries + Periodontitis (n=991)** | **FDR** |
| --- | --- | --- | --- | --- | --- | --- | --- |
| Dietary indices | |  |  |  |  |  |  |
|  | HEI2020 | 52.58 (44.37 - 61.10) | 54.51 (46.14 - 63.01) | 47.89 (40.36 - 58.66) | 52.58 (44.76 - 60.83) | 47.86 (40.83 - 55.94) | **<0.001** |
|  | DASH | 23.00 (19.50 - 26.50) | 24.00 (20.50 - 27.50) | 21.00 (17.50 - 25.00) | 22.50 (19.50 - 26.50) | 20.00 (17.50 - 23.00) | **<0.001** |
|  | MED | 3.50 (2.50 - 4.50) | 4.00 (3.00 - 5.00) | 3.00 (2.00 - 4.00) | 3.50 (2.50 - 4.50) | 3.00 (2.00 - 4.00) | **<0.001** |
|  | DII | 1.85 (0.45 - 3.06) | 1.56 (0.22 - 2.90) | 2.44 (0.65 - 3.39) | 1.86 (0.57 - 3.03) | 2.42 (1.13 - 3.53) | **<0.001** |
| Number of foods reported | | 16.50 (13.00 - 20.00) | 17.50 (14.50 - 21.00) | 15.50 (12.50 - 18.50) | 16.00 (13.00 - 19.50) | 14.50 (11.50 - 18.00) | **<0.001** |
| Food patterns | |  |  |  |  |  |  |
|  | Citrus, melons, and berries (cup eq./1000 kcal) | 0.00 (0.00 - 0.14) | 0.01 (0.00 - 0.17) | 0.00 (0.00 - 0.08) | 0.00 (0.00 - 0.14) | 0.00 (0.00 - 0.01) | **<0.001** |
|  | Other fruits (cup eq./1000 kcal) | 0.14 (0.00 - 0.41) | 0.17 (0.01 - 0.45) | 0.04 (0.00 - 0.31) | 0.16 (0.00 - 0.42) | 0.05 (0.00 - 0.31) | **<0.001** |
|  | Fruit juice (cup eq./1000 kcal) | 0.00 (0.00 - 0.14) | 0.00 (0.00 - 0.13) | 0.00 (0.00 - 0.16) | 0.00 (0.00 - 0.18) | 0.00 (0.00 - 0.10) | 0.247 |
|  | Total fruit (cup eq./1000 kcal) | 0.37 (0.08 - 0.76) | 0.40 (0.12 - 0.83) | 0.28 (0.02 - 0.53) | 0.39 (0.13 - 0.80) | 0.25 (0.02 - 0.60) | **<0.001** |
|  | Dark green vegetables (cup eq./1000 kcal) | 0.00 (0.00 - 0.12) | 0.00 (0.00 - 0.16) | 0.00 (0.00 - 0.04) | 0.00 (0.00 - 0.09) | 0.00 (0.00 - 0.02) | **<0.001** |
|  | Tomatoes (cup eq./1000 kcal) | 0.11 (0.04 - 0.21) | 0.12 (0.04 - 0.22) | 0.09 (0.03 - 0.18) | 0.10 (0.03 - 0.21) | 0.11 (0.03 - 0.20) | 0.168 |
|  | Other red and orange vegetables (cup eq./1000 kcal) | 0.01 (0.00 - 0.07) | 0.01 (0.00 - 0.09) | 0.00 (0.00 - 0.06) | 0.01 (0.00 - 0.06) | 0.00 (0.00 - 0.03) | **<0.001** |
|  | Total red and orange vegetables (cup eq./1000 kcal) | 0.16 (0.07 - 0.29) | 0.18 (0.08 - 0.31) | 0.12 (0.06 - 0.28) | 0.14 (0.06 - 0.29) | 0.14 (0.06 - 0.25) | **<0.001** |
|  | Potatoes (white potatoes) (cup eq./1000 kcal) | 0.10 (0.00 - 0.26) | 0.09 (0.00 - 0.24) | 0.09 (0.00 - 0.25) | 0.11 (0.00 - 0.26) | 0.13 (0.00 - 0.29) | 0.066 |
|  | Other starchy vegetables (cup eq./1000 kcal) | 0.00 (0.00 - 0.04) | 0.00 (0.00 - 0.04) | 0.00 (0.00 - 0.04) | 0.00 (0.00 - 0.04) | 0.00 (0.00 - 0.02) | 0.145 |
|  | Total starchy vegetables (cup eq./1000 kcal) | 0.15 (0.00 - 0.31) | 0.14 (0.00 - 0.30) | 0.14 (0.00 - 0.30) | 0.15 (0.00 - 0.31) | 0.17 (0.00 - 0.34) | 0.326 |
|  | Other vegetables (cup eq./1000 kcal) | 0.23 (0.10 - 0.43) | 0.25 (0.12 - 0.45) | 0.18 (0.06 - 0.35) | 0.24 (0.09 - 0.42) | 0.17 (0.06 - 0.37) | **<0.001** |
|  | Total vegetables (cup eq./1000 kcal) | 0.73 (0.46 - 1.10) | 0.80 (0.50 - 1.15) | 0.62 (0.36 - 0.91) | 0.70 (0.44 - 1.14) | 0.62 (0.39 - 0.93) | **<0.001** |
|  | Beans and peas computed as vegetables (cup eq./1000 kcal) | 0.00 (0.00 - 0.07) | 0.00 (0.00 - 0.07) | 0.00 (0.00 - 0.06) | 0.00 (0.00 - 0.07) | 0.00 (0.00 - 0.10) | 0.490 |
|  | Whole grains (oz. eq./1000 kcal) | 0.31 (0.00 - 0.79) | 0.38 (0.07 - 0.88) | 0.22 (0.00 - 0.57) | 0.30 (0.00 - 0.76) | 0.13 (0.00 - 0.52) | **<0.001** |
|  | Refined grains (oz. eq./1000 kcal) | 2.52 (1.81 - 3.35) | 2.49 (1.79 - 3.32) | 2.47 (1.88 - 3.24) | 2.57 (1.82 - 3.38) | 2.56 (1.87 - 3.46) | 0.446 |
|  | Total grains (oz. eq./1000 kcal) | 3.09 (2.34 - 3.86) | 3.12 (2.40 - 3.89) | 3.03 (2.26 - 3.68) | 3.12 (2.33 - 3.86) | 2.96 (2.27 - 3.85) | 0.247 |
|  | Meat (oz. eq./1000 kcal) | 0.55 (0.00 - 1.19) | 0.45 (0.00 - 1.09) | 0.65 (0.00 - 1.31) | 0.61 (0.00 - 1.29) | 0.61 (0.00 - 1.35) | **0.034** |
|  | Cured meat (oz. eq./1000 kcal) | 0.25 (0.00 - 0.70) | 0.25 (0.00 - 0.71) | 0.20 (0.00 - 0.68) | 0.26 (0.00 - 0.70) | 0.26 (0.00 - 0.72) | 0.682 |
|  | Organ meat (oz. eq./1000 kcal) | 0.00 (0.00 - 0.00) | 0.00 (0.00 - 0.00) | 0.00 (0.00 - 0.00) | 0.00 (0.00 - 0.00) | 0.00 (0.00 - 0.00) | 0.490 |
|  | Poultry (oz. eq./1000 kcal) | 0.49 (0.00 - 1.14) | 0.53 (0.00 - 1.14) | 0.47 (0.00 - 1.09) | 0.44 (0.00 - 1.11) | 0.40 (0.00 - 1.18) | 0.210 |
|  | Seafood high in n-3 fatty acids (oz. eq./1000 kcal) | 0.00 (0.00 - 0.00) | 0.00 (0.00 - 0.00) | 0.00 (0.00 - 0.00) | 0.00 (0.00 - 0.00) | 0.00 (0.00 - 0.00) | 0.423 |
|  | Seafood low in n-3 fatty acids (oz. eq./1000 kcal) | 0.00 (0.00 - 0.12) | 0.00 (0.00 - 0.14) | 0.00 (0.00 - 0.00) | 0.00 (0.00 - 0.19) | 0.00 (0.00 - 0.00) | 0.343 |
|  | Total meat, poultry, and seafood (oz. eq./1000 kcal) | 2.15 (1.44 - 3.08) | 2.09 (1.38 - 2.98) | 2.30 (1.36 - 3.19) | 2.24 (1.47 - 3.21) | 2.20 (1.52 - 3.19) | 0.146 |
|  | Eggs (oz. eq./1000 kcal) | 0.12 (0.02 - 0.42) | 0.11 (0.02 - 0.40) | 0.12 (0.02 - 0.44) | 0.14 (0.02 - 0.46) | 0.12 (0.02 - 0.43) | 0.268 |
|  | Soybean products (oz. eq./1000 kcal) | 0.00 (0.00 - 0.00) | 0.00 (0.00 - 0.00) | 0.00 (0.00 - 0.00) | 0.00 (0.00 - 0.00) | 0.00 (0.00 - 0.00) | 0.083 |
|  | Nuts and seeds (oz. eq./1000 kcal) | 0.09 (0.00 - 0.49) | 0.15 (0.00 - 0.63) | 0.00 (0.00 - 0.29) | 0.07 (0.00 - 0.43) | 0.00 (0.00 - 0.14) | **<0.001** |
|  | Beans and peas computed as protein foods (oz. eq./1000 kcal) | 0.00 (0.00 - 0.30) | 0.00 (0.00 - 0.29) | 0.00 (0.00 - 0.24) | 0.00 (0.00 - 0.30) | 0.00 (0.00 - 0.42) | 0.490 |
|  | Total protein foods (oz. eq./1000 kcal) | 2.83 (2.02 - 3.83) | 2.82 (2.03 - 3.80) | 2.86 (2.03 - 3.96) | 2.88 (2.09 - 3.89) | 2.68 (1.89 - 3.79) | 0.474 |
|  | Milk (cup eq./1000 kcal) | 0.21 (0.06 - 0.50) | 0.22 (0.06 - 0.51) | 0.22 (0.06 - 0.52) | 0.23 (0.07 - 0.51) | 0.17 (0.04 - 0.40) | **0.023** |
|  | Yogurt (cup eq./1000 kcal) | 0.00 (0.00 - 0.00) | 0.00 (0.00 - 0.01) | 0.00 (0.00 - 0.00) | 0.00 (0.00 - 0.00) | 0.00 (0.00 - 0.00) | **<0.001** |
|  | Cheese (cup eq./1000 kcal) | 0.28 (0.08 - 0.53) | 0.29 (0.09 - 0.56) | 0.31 (0.08 - 0.55) | 0.24 (0.03 - 0.49) | 0.27 (0.07 - 0.52) | 0.119 |
|  | Total dairy (cup eq./1000 kcal) | 0.69 (0.39 - 1.02) | 0.72 (0.43 - 1.04) | 0.70 (0.36 - 1.11) | 0.67 (0.39 - 0.98) | 0.60 (0.33 - 0.92) | **0.026** |
|  | Oils (g/1000 kcal) | 11.59 (7.91 - 15.96) | 12.22 (8.63 - 16.85) | 11.13 (7.47 - 14.97) | 10.88 (7.51 - 15.15) | 10.55 (6.83 - 14.50) | **<0.001** |
|  | Solid fats (g/1000 kcal) | 15.84 (11.39 - 20.49) | 15.70 (11.08 - 20.02) | 15.96 (11.73 - 20.37) | 15.86 (11.53 - 21.50) | 16.09 (11.70 - 20.52) | 0.236 |
|  | Added sugars (tsp. eq./1000 kcal) | 6.57 (4.06 - 9.87) | 6.34 (3.98 - 9.43) | 8.54 (5.27 - 11.94) | 6.14 (3.72 - 9.35) | 7.76 (4.70 - 12.30) | **<0.001** |
|  | Alcoholic drinks (no. of drinks/1000 kcal) | 0.00 (0.00 - 0.36) | 0.00 (0.00 - 0.38) | 0.00 (0.00 - 0.29) | 0.00 (0.00 - 0.39) | 0.00 (0.00 - 0.17) | **0.004** |
| Nutrients | |  |  |  |  |  |  |
|  | Protein intake (gm/1000 kcal) | 38.93 (33.21 - 45.92) | 39.32 (33.54 - 45.69) | 38.38 (31.78 - 46.12) | 39.42 (33.68 - 46.60) | 37.31 (31.57 - 45.43) | 0.092 |
|  | Carbohydrate intake (gm/1000 kcal) | 120.61 (105.84 - 135.53) | 120.76 (105.99 - 134.33) | 121.94 (107.65 - 137.12) | 118.09 (103.78 - 134.08) | 123.06 (106.43 - 139.23) | 0.109 |
|  | Total sugars intake (gm/1000 kcal) | 49.72 (36.59 - 63.74) | 49.34 (36.46 - 62.14) | 54.70 (42.41 - 68.02) | 48.56 (34.99 - 63.59) | 51.62 (36.73 - 69.84) | **0.004** |
|  | Dietary fiber intake (gm/1000 kcal) | 8.40 (6.27 - 11.00) | 8.85 (6.85 - 11.35) | 7.01 (4.89 - 9.56) | 8.34 (6.35 - 10.90) | 7.27 (5.63 - 9.76) | **<0.001** |
|  | Total fat intake (gm/1000 kcal) | 38.02 (32.66 - 42.88) | 38.36 (33.35 - 42.90) | 36.63 (32.36 - 42.23) | 38.00 (32.11 - 43.13) | 37.13 (31.53 - 42.06) | 0.112 |
|  | Total saturated fatty acids (gm/1000 kcal) | 11.92 (9.77 - 14.27) | 11.86 (9.77 - 14.12) | 12.06 (9.93 - 14.12) | 12.08 (9.81 - 14.65) | 11.85 (9.53 - 14.12) | 0.596 |
|  | Total monounsaturated fatty acids (gm/1000 kcal) | 13.17 (11.01 - 15.36) | 13.29 (11.15 - 15.36) | 12.79 (10.66 - 14.61) | 13.27 (11.00 - 15.61) | 12.81 (10.73 - 14.98) | 0.057 |
|  | Total polyunsaturated fatty acids (gm/1000 kcal) | 8.71 (6.96 - 10.60) | 9.01 (7.21 - 10.88) | 8.35 (6.56 - 10.25) | 8.36 (6.75 - 10.37) | 8.40 (6.55 - 10.04) | **<0.001** |
|  | Cholesterol (mg/1000 kcal) | 124.36 (87.30 - 172.14) | 119.44 (83.01 - 164.96) | 126.91 (88.26 - 177.76) | 131.40 (92.83 - 181.31) | 126.73 (87.05 - 178.57) | **0.002** |
|  | Alcohol (gm/1000 kcal) | 0.00 (0.00 - 5.07) | 0.00 (0.00 - 5.35) | 0.00 (0.00 - 4.16) | 0.00 (0.00 - 5.37) | 0.00 (0.00 - 2.35) | **0.003** |
|  | Retinol (mcg/1000 kcal) | 181.02 (113.28 - 261.53) | 185.67 (118.47 - 267.03) | 171.99 (106.85 - 258.23) | 187.42 (116.33 - 262.09) | 157.53 (94.88 - 229.87) | **0.002** |
|  | Vitamin A, RAE (mcg/1000 kcal) | 274.87 (190.27 - 419.97) | 305.99 (207.78 - 445.35) | 244.57 (170.08 - 383.55) | 270.35 (190.60 - 416.21) | 222.63 (149.79 - 316.29) | **<0.001** |
|  | Vitamin D (D2+D3) (mcg/1000 kcal) | 1.78 (0.94 - 2.91) | 1.76 (0.94 - 2.82) | 1.75 (0.89 - 3.22) | 1.90 (1.00 - 3.08) | 1.60 (0.92 - 2.76) | 0.064 |
|  | Vitamin E as alpha-tocopherol (mg/1000 kcal) | 3.88 (2.99 - 5.21) | 4.11 (3.20 - 5.64) | 3.52 (2.61 - 4.52) | 3.88 (2.92 - 5.06) | 3.34 (2.64 - 4.47) | **<0.001** |
|  | Added alpha-tocopherol (Vitamin E) (mg/1000 kcal) | 0.00 (0.00 - 0.00) | 0.00 (0.00 - 0.00) | 0.00 (0.00 - 0.00) | 0.00 (0.00 - 0.00) | 0.00 (0.00 - 0.00) | **<0.001** |
|  | Vitamin K (mcg/1000 kcal) | 41.22 (26.75 - 74.99) | 48.45 (28.93 - 86.34) | 31.29 (22.30 - 54.54) | 39.96 (26.53 - 70.68) | 33.19 (22.14 - 49.74) | **<0.001** |
|  | Alpha-carotene (mcg/1000 kcal) | 50.81 (14.63 - 266.82) | 63.84 (18.04 - 357.14) | 22.75 (7.03 - 140.68) | 51.38 (15.20 - 216.27) | 35.79 (9.83 - 144.59) | **<0.001** |
|  | Beta-carotene (mcg/1000 kcal) | 656.57 (263.03 - 1660.99) | 839.06 (324.18 - 2005.18) | 364.11 (164.89 - 1125.53) | 603.28 (259.94 - 1494.03) | 408.99 (175.54 - 912.27) | **<0.001** |
|  | Beta-cryptoxanthin (mcg/1000 kcal) | 21.39 (7.95 - 52.19) | 22.11 (8.31 - 53.04) | 18.62 (7.63 - 46.46) | 23.54 (9.08 - 54.86) | 16.98 (5.62 - 45.74) | **0.022** |
|  | Thiamin (Vitamin B1) (mg/1000 kcal) | 0.76 (0.62 - 0.92) | 0.76 (0.63 - 0.93) | 0.71 (0.60 - 0.88) | 0.76 (0.63 - 0.93) | 0.73 (0.58 - 0.93) | **0.027** |
|  | Riboflavin (Vitamin B2) (mg/1000 kcal) | 0.99 (0.79 - 1.23) | 1.02 (0.82 - 1.25) | 0.95 (0.74 - 1.23) | 0.99 (0.81 - 1.22) | 0.93 (0.73 - 1.14) | **<0.001** |
|  | Niacin (mg/1000 kcal) | 11.80 (9.80 - 14.50) | 11.81 (9.90 - 14.64) | 11.38 (9.51 - 14.07) | 12.03 (9.75 - 14.45) | 11.61 (9.76 - 14.41) | 0.474 |
|  | Vitamin B6 (mg/1000 kcal) | 0.96 (0.76 - 1.25) | 0.98 (0.78 - 1.27) | 0.90 (0.70 - 1.22) | 0.98 (0.78 - 1.26) | 0.91 (0.71 - 1.18) | **0.022** |
|  | Total Folate (mcg/1000 kcal) | 187.04 (146.26 - 243.28) | 193.41 (154.44 - 252.54) | 172.91 (134.88 - 217.34) | 190.00 (146.09 - 245.25) | 167.46 (131.17 - 222.19) | **<0.001** |
|  | Folic acid (mcg/1000 kcal) | 72.60 (46.66 - 113.81) | 73.54 (46.22 - 115.22) | 68.44 (47.68 - 102.28) | 73.57 (48.47 - 116.82) | 67.42 (45.75 - 105.79) | 0.216 |
|  | Food folate (mcg/1000 kcal) | 103.19 (78.74 - 135.85) | 109.19 (82.95 - 142.01) | 92.16 (71.19 - 115.65) | 103.46 (80.85 - 136.51) | 88.94 (68.68 - 118.75) | **<0.001** |
|  | Folate DFE (mcg/1000 kcal) | 241.27 (184.07 - 317.93) | 247.88 (192.01 - 330.02) | 218.38 (173.52 - 282.68) | 244.00 (183.12 - 323.75) | 215.37 (164.84 - 293.51) | **<0.001** |
|  | Vitamin B12 (mcg/1000 kcal) | 2.05 (1.39 - 2.90) | 2.05 (1.39 - 2.86) | 1.94 (1.45 - 3.03) | 2.10 (1.44 - 3.00) | 1.98 (1.31 - 2.84) | 0.278 |
|  | Added vitamin B12 (mcg/1000 kcal) | 0.00 (0.00 - 0.67) | 0.00 (0.00 - 0.72) | 0.00 (0.00 - 0.65) | 0.00 (0.00 - 0.64) | 0.00 (0.00 - 0.50) | 0.072 |
|  | Vitamin C (mg/1000 kcal) | 32.55 (15.61 - 58.95) | 34.85 (18.48 - 60.30) | 24.12 (10.99 - 47.77) | 33.79 (16.13 - 62.38) | 24.95 (10.14 - 50.70) | **<0.001** |
|  | Total choline (mg/1000 kcal) | 153.93 (124.99 - 191.44) | 151.40 (124.53 - 188.61) | 153.93 (123.86 - 191.42) | 160.35 (130.32 - 199.38) | 150.00 (121.74 - 188.25) | 0.036 |
|  | Calcium (mg/1000 kcal) | 448.42 (341.34 - 568.89) | 456.59 (357.57 - 584.25) | 424.88 (316.55 - 557.34) | 444.46 (342.06 - 555.56) | 418.17 (301.48 - 540.45) | **0.001** |
|  | Phosphorus (mg/1000 kcal) | 661.94 (574.07 - 763.92) | 670.30 (584.94 - 770.53) | 643.50 (555.24 - 758.62) | 664.05 (571.73 - 771.00) | 628.95 (539.24 - 733.62) | **0.001** |
|  | Magnesium (mg/1000 kcal) | 147.18 (119.88 - 179.79) | 151.91 (126.94 - 185.77) | 137.17 (108.16 - 165.29) | 145.77 (119.00 - 178.91) | 129.39 (107.80 - 164.16) | **<0.001** |
|  | Sodium (mg/1000 kcal) | 1651.46 (1419.23 - 1919.91) | 1647.13 (1434.21 - 1913.97) | 1621.61 (1389.37 - 1881.41) | 1674.64 (1427.76 - 1953.22) | 1636.72 (1349.80 - 1912.96) | 0.092 |
|  | Potassium (mg/1000 kcal) | 1321.65 (1100.45 - 1580.66) | 1351.60 (1131.21 - 1607.39) | 1212.05 (982.23 - 1517.25) | 1333.33 (1106.52 - 1599.39) | 1229.10 (1018.64 - 1493.00) | **<0.001** |
|  | Iron (mg/1000 kcal) | 6.77 (5.59 - 8.49) | 6.86 (5.67 - 8.53) | 6.20 (5.16 - 7.76) | 6.89 (5.75 - 8.76) | 6.37 (5.19 - 7.95) | **0.003** |
|  | Zinc (mg/1000 kcal) | 5.14 (4.25 - 6.33) | 5.17 (4.33 - 6.39) | 5.02 (4.16 - 6.09) | 5.23 (4.32 - 6.33) | 4.91 (3.96 - 6.03) | **0.029** |
|  | Copper (mg/1000 kcal) | 0.59 (0.48 - 0.74) | 0.62 (0.50 - 0.77) | 0.52 (0.42 - 0.67) | 0.59 (0.48 - 0.75) | 0.54 (0.42 - 0.68) | **<0.001** |
|  | Selenium (mcg/1000 kcal) | 54.15 (45.48 - 64.54) | 54.07 (45.72 - 63.96) | 51.94 (45.42 - 65.58) | 55.25 (45.74 - 65.59) | 53.24 (43.83 - 63.83) | 0.423 |
|  | Lycopene (mcg/1000 kcal) | 1383.72 (442.08 - 3302.51) | 1458.80 (481.56 - 3414.47) | 1367.28 (356.71 - 2907.24) | 1312.66 (394.45 - 3115.65) | 1347.79 (465.39 - 3178.89) | 0.343 |
|  | Lutein + zeaxanthin (mcg/1000 kcal) | 466.27 (259.57 - 944.20) | 531.57 (302.13 - 1196.12) | 345.28 (214.50 - 649.33) | 463.11 (242.06 - 854.89) | 356.27 (203.08 - 585.60) | **<0.001** |
|  | Caffeine (mg/1000 kcal) | 65.22 (23.34 - 123.28) | 66.78 (24.11 - 122.87) | 60.31 (20.51 - 119.65) | 61.45 (21.95 - 119.51) | 70.07 (26.52 - 133.38) | 0.326 |
|  | Theobromine (mg/1000 kcal) | 8.09 (0.00 - 24.57) | 10.02 (0.00 - 26.73) | 6.66 (0.00 - 21.67) | 6.27 (0.00 - 21.47) | 5.10 (0.00 - 21.35) | **0.029** |
|  | Total plain water drank yesterday (gm/1000 kcal) | 450.63 (192.20 - 836.16) | 504.58 (239.73 - 873.14) | 375.00 (138.14 - 692.09) | 409.80 (167.34 - 799.11) | 348.23 (105.94 - 730.48) | **<0.001** |
|  | Total tap water drank yesterday (gm/1000 kcal) | 198.62 (0.00 - 547.68) | 272.37 (43.01 - 651.19) | 82.31 (0.00 - 367.12) | 146.91 (0.00 - 445.17) | 82.87 (0.00 - 407.59) | **<0.001** |
|  | Total bottled water drank (gm/1000 kcal) | 0.00 (0.00 - 278.95) | 0.00 (0.00 - 246.71) | 30.39 (0.00 - 373.95) | 0.00 (0.00 - 309.98) | 0.00 (0.00 - 309.71) | 0.179 |
|  | Moisture (gm/1000 kcal) | 1379.02 (1063.15 - 1833.69) | 1418.12 (1107.46 - 1848.38) | 1320.71 (986.25 - 1757.67) | 1346.85 (1047.09 - 1779.21) | 1323.08 (1001.75 - 1838.31) | **0.029** |
|  | SFA 4:0 (Butanoic) (gm/1000 kcal) | 0.21 (0.13 - 0.33) | 0.22 (0.14 - 0.34) | 0.22 (0.13 - 0.31) | 0.22 (0.12 - 0.34) | 0.19 (0.11 - 0.30) | **0.031** |
|  | SFA 6:0 (Hexanoic) (gm/1000 kcal) | 0.13 (0.08 - 0.19) | 0.13 (0.08 - 0.20) | 0.13 (0.08 - 0.19) | 0.13 (0.07 - 0.20) | 0.11 (0.07 - 0.18) | **0.019** |
|  | SFA 8:0 (Octanoic) (gm/1000 kcal) | 0.10 (0.07 - 0.15) | 0.10 (0.07 - 0.15) | 0.11 (0.07 - 0.16) | 0.10 (0.06 - 0.16) | 0.09 (0.06 - 0.15) | 0.103 |
|  | SFA 10:0 (Decanoic) (gm/1000 kcal) | 0.21 (0.13 - 0.30) | 0.21 (0.14 - 0.31) | 0.21 (0.14 - 0.30) | 0.21 (0.12 - 0.31) | 0.18 (0.12 - 0.28) | **0.008** |
|  | SFA 12:0 (Dodecanoic) (gm/1000 kcal) | 0.29 (0.17 - 0.45) | 0.29 (0.18 - 0.47) | 0.30 (0.18 - 0.46) | 0.28 (0.17 - 0.45) | 0.26 (0.15 - 0.43) | 0.138 |
|  | SFA 14:0 (Tetradecanoic) (gm/1000 kcal) | 0.95 (0.64 - 1.31) | 0.97 (0.65 - 1.32) | 0.98 (0.68 - 1.30) | 0.96 (0.64 - 1.31) | 0.87 (0.63 - 1.24) | 0.181 |
|  | SFA 16:0 (Hexadecanoic) (gm/1000 kcal) | 6.44 (5.42 - 7.65) | 6.40 (5.40 - 7.55) | 6.42 (5.51 - 7.65) | 6.61 (5.44 - 7.78) | 6.40 (5.34 - 7.69) | 0.485 |
|  | SFA 18:0 (Octadecanoic) (gm/1000 kcal) | 2.92 (2.37 - 3.52) | 2.89 (2.34 - 3.52) | 2.94 (2.47 - 3.50) | 2.97 (2.40 - 3.60) | 2.93 (2.34 - 3.49) | 0.486 |
|  | MFA 16:1 (Hexadecenoic) (gm/1000 kcal) | 0.46 (0.35 - 0.60) | 0.45 (0.33 - 0.58) | 0.46 (0.36 - 0.60) | 0.49 (0.37 - 0.64) | 0.48 (0.36 - 0.64) | **<0.001** |
|  | MFA 18:1 (Octadecenoic) (gm/1000 kcal) | 12.08 (10.02 - 14.19) | 12.21 (10.25 - 14.28) | 11.55 (9.89 - 13.40) | 12.25 (9.98 - 14.53) | 11.66 (9.59 - 13.73) | **0.007** |
|  | MFA 20:1 (Eicosenoic) (gm/1000 kcal) | 0.13 (0.09 - 0.17) | 0.12 (0.09 - 0.17) | 0.13 (0.10 - 0.17) | 0.13 (0.09 - 0.17) | 0.13 (0.09 - 0.17) | 0.534 |
|  | MFA 22:1 (Docosenoic) (gm/1000 kcal) | 0.01 (0.00 - 0.01) | 0.01 (0.00 - 0.02) | 0.01 (0.00 - 0.02) | 0.01 (0.00 - 0.01) | 0.01 (0.00 - 0.02) | 0.172 |
|  | PFA 18:2 (Octadecadienoic) (gm/1000 kcal) | 7.72 (6.08 - 9.43) | 7.98 (6.30 - 9.63) | 7.41 (5.82 - 9.16) | 7.35 (5.89 - 9.20) | 7.31 (5.71 - 8.95) | **<0.001** |
|  | PFA 18:3 (Octadecatrienoic) (gm/1000 kcal) | 0.78 (0.60 - 1.01) | 0.81 (0.62 - 1.05) | 0.73 (0.58 - 0.94) | 0.76 (0.59 - 0.99) | 0.74 (0.56 - 0.95) | **<0.001** |
|  | PFA 18:4 (Octadecatetraenoic) (gm/1000 kcal) | 0.00 (0.00 - 0.00) | 0.00 (0.00 - 0.00) | 0.00 (0.00 - 0.00) | 0.00 (0.00 - 0.00) | 0.00 (0.00 - 0.00) | **0.035** |
|  | PFA 20:4 (Eicosatetraenoic) (gm/1000 kcal) | 0.06 (0.04 - 0.09) | 0.06 (0.04 - 0.09) | 0.07 (0.04 - 0.10) | 0.07 (0.04 - 0.10) | 0.07 (0.04 - 0.10) | **0.008** |
|  | PFA 20:5 (Eicosapentaenoic) (gm/1000 kcal) | 0.00 (0.00 - 0.01) | 0.00 (0.00 - 0.01) | 0.00 (0.00 - 0.01) | 0.00 (0.00 - 0.01) | 0.00 (0.00 - 0.01) | 0.444 |
|  | PFA 22:5 (Docosapentaenoic) (gm/1000 kcal) | 0.01 (0.01 - 0.01) | 0.01 (0.01 - 0.01) | 0.01 (0.01 - 0.01) | 0.01 (0.01 - 0.01) | 0.01 (0.01 - 0.01) | 0.057 |
|  | PFA 22:6 (Docosahexaenoic) (gm/1000 kcal) | 0.01 (0.00 - 0.03) | 0.01 (0.00 - 0.03) | 0.01 (0.00 - 0.03) | 0.02 (0.00 - 0.04) | 0.01 (0.00 - 0.03) | 0.162 |

*Note:*

*HEI2020, Healthy Eating Index 2020; DASH, Dietary Approaches to Stop Hypertension Index; MED, alternate Mediterranean Diet Score; DII, Dietary Inflammatory Index; IQR, interquartile range; FDR, false discovery rate.*

*All included participants had two reliable 24-hour dietary recalls. Dietary indices, the number of foods reported, and food and nutrient intake variables were calculated as the average of the two recall days. Food and nutrient intake variables were energy-adjusted using the per 1,000 kcal method.* *The food and nutrient intake variables listed in this table were also used in the PCA analyses.*

*All estimates accounted for the NHANES complex sampling design, including sampling weights, strata, and primary sampling units. Variables are presented as survey-weighted medians and interquartile ranges (IQRs). P values were calculated using survey-weighted Kruskal-Wallis rank tests. Benjamini-Hochberg FDR correction was applied for multiple comparisons.*
